# Sex-Biased Control of Inflammation and Metabolism by a Mitochondrial Nod-Like Receptor

**DOI:** 10.3389/fimmu.2022.882867

**Published:** 2022-05-16

**Authors:** Tiia Snäkä, Amel Bekkar, Chantal Desponds, Florence Prével, Stéphanie Claudinot, Nathalie Isorce, Filipa Teixeira, Coline Grasset, Ioannis Xenarios, Isabel C. Lopez-Mejia, Lluis Fajas, Nicolas Fasel

**Affiliations:** ^1^ Department of Biochemistry, University of Lausanne, Epalinges, Switzerland; ^2^ Agora Center, Center Hospitalier Universitaire (CHUV), Lausanne, Switzerland; ^3^ Center for Integrative Genomics, University of Lausanne, Lausanne, Switzerland

**Keywords:** inflammation, innate immunity, metabolism, sex, nod-like receptor X1

## Abstract

Mitochondria regulate steroid hormone synthesis, and in turn sex hormones regulate mitochondrial function for maintaining cellular homeostasis and controlling inflammation. This crosstalk can explain sex differences observed in several pathologies such as in metabolic or inflammatory disorders. Nod-like receptor X1 (NLRX1) is a mitochondria-associated innate receptor that could modulate metabolic functions and attenuates inflammatory responses. Here, we showed that in an infectious model with the human protozoan parasite, *Leishmania guyanensis*, NLRX1 attenuated inflammation in females but not in male mice. Analysis of infected female and male bone marrow derived macrophages showed both sex- and genotype-specific differences in both inflammatory and metabolic profiles with increased type I interferon production, mitochondrial respiration, and glycolytic rate in *Nlrx1*-deficient female BMDMs in comparison to wild-type cells, while no differences were observed between males. Transcriptomics of female and male BMDMs revealed an altered steroid hormone signaling in *Nlrx1*-deficient cells, and a “masculinization” of *Nlrx1*-deficient female BMDMs. Thus, our findings suggest that NLRX1 prevents uncontrolled inflammation and metabolism in females and therefore may contribute to the sex differences observed in infectious and inflammatory diseases.

## Introduction

Different factors including XY-encoded genes and sex hormones contribute to sex-dependent variations in the incidence of different infectious and inflammatory diseases (1). Transcriptional analysis of unstimulated female and male immune cells has revealed sex-specific gene expression patterns, with differences mainly in type I interferon (IFN)-response genes that were enriched in females (2–4). In addition, immune cells express receptors for sex hormones: estrogens (17-β-estradiol), androgens (testosterone) and progesterone. These hormones are modulators of immune cells and contribute to differences in cell activation and functionality (5–7). Binding of estrogens to their nuclear receptors, estrogen receptor alpha and beta (ERα and ERβ, respectively) promotes or dampens immune signaling in innate immune cells in a dose-dependent manner. While physiological levels of estrogens tend to promote type I IFN responses, higher doses are immunosuppressive (1, 5, 8). Moreover, estrogens play a key role in the resolution of inflammation and cutaneous repair by promoting anti-inflammatory macrophage activation (9–11).

To respond to infection and cellular damage, immune cells are able to adapt their functional profiles not only by activation of different transcriptional profiles but also by engaging specific metabolic pathways. Indeed, the regulation of energy metabolism is crucial for innate immune cell function and for example plays a major role in macrophage activation and polarization to either pro-inflammatory M1 or anti-inflammatory M2 macrophage subtypes allowing adaptation to different environments. While M1 macrophages are characterized by a high glycolytic rate and play a role in pathogen clearance, M2 macrophages rely on mitochondrial oxidative phosphorylation (OXPHOS) and promote tissue repair (12–14). Mitochondria are key organelles in energy metabolism and sex- and tissue-specific differences in mitochondrial function and morphology have been reported (15–17). Mitochondrial enzymes play a key role in sex hormone biosynthesis, and in turn sex hormones, mainly estrogens, regulate mitochondrial function and morphology *via* nuclear or mitochondrial ERs to promote mitochondrial metabolism (18, 19).

Nod-like receptor X1 (NLRX1) is a unique mitochondrial NOD-like receptor (NLR) implicated in the control of inflammation and metabolism in both infectious and inflammatory diseases. It was first described as a traditional RNA-binding pathogen recognition receptor (PRR) involved in mitochondrial antiviral immunity mainly by attenuating type I IFN or nuclear factor-κB (NF-κB) signaling (20–23). However, recent studies have shown that NLRX1 plays an important role in the control of inflammation in several models of cancer and tissue injury, independently of its role in pathogen recognition (24–27). In addition, due to its localization at the mitochondria, the central hub of metabolism and immunity, several studies suggest a role for NLRX1 in the maintenance of mitochondrial physiology, function, and reactive oxygen species (mtROS) production following infection or injury (28–31). Interestingly, sex differences in all these aspects have been described, however, no studies have reported a potential link to an NLR.

To investigate the role of NLRX1, we used an experimental murine model based on a protozoan parasite, *Leishmania guyanensis* (*Lgy*), inducing cutaneous lesions at the site of the infection. This causative agent of human cutaneous leishmaniasis can induce a more exacerbated hyperinflammatory form of the disease when carrying in its cytoplasm an endosymbiont virus, *Leishmania* RNA Virus 1 (LRV1) with a double-stranded viral RNA as genome (32–35). Upon phagocytosis of the parasite into macrophages, the viral double stranded RNA (dsRNA) is recognized by the macrophage endosomal Toll-like receptor 3 (TLR3) and induces a strong type I IFN mediated antiviral response and NF-κB mediated pro-inflammatory cytokines such as interleukin 6 (IL-6) and as tumor-necrosis factor α (TNFα) leading to an exacerbated disease outcome (36, 37). This experimental model allowed us to investigate not only the inflammatory response in *in vitro* infected macrophages but also the *in vivo* development of exacerbated lesions in mice. Here, we showed that NLRX1 controlled viral mediated inflammation and metabolism in a sex-dependent manner.

## Materials and Methods

### Ethics Statement

All animal experimentation protocols described in this study were approved by the Swiss Federal Veterinary Office (SFVO), under authorization numbers VD2113.2 and VD3551. Animal handling and experimental procedures were undertaken with strict adherence to the ethical guidelines given by the SFVO and under inspection by the Department of Security and Environment of the State of Vaud, Switzerland.

### Mice

Wild-type (WT) (C57BL/6JOlaHsd) mice were purchased from Envigo (Netherlands) and *Nlrx1*-deficient mice (*Nlrx1^-/-^)* (B6.129-Nlrx1tm1 Tsc) were generated previously by replacing the first four coding exons with a neomycin cassette, that was later removed (38). The mice were previously backcrossed onto the C57BL/6JOlaHsd background for at least 5 generations. Mice were genotyped by PCR using tissue-isolated genomic DNA using the KAPA Mouse Genotyping Kit (KAPA Biosystems). Mice were maintained at the animal facility of the Center for Immunity and Immunology Lausanne (CIIL) (Switzerland) in a pathogen-free environment. Males and females (6-9 weeks old) were used for experiments. *In vivo* experiments were performed at a biosafety level 2 (BSL-2) animal facility at the CIIL. Cages were enriched with one igloo, two carboard tubes, one wood stick, and tissues. Experiments were performed after one week of acclimation in the BSL-2 animal facility. Food (SAFE or KLIBA NAGAF) and water were provided ad libitum. Light cycle was maintained at 13 hours light and 11 hours darkness, temperature was set at 21°C ± 2 and humidity was kept at 55% ± 10. The oligonucleotides used for genotyping of *Nlrx1-*deficient mice were:

*Nlrx1* “WT For”: 5’-TTA GAC TGG TGT TAC GGG AGA CTG-3’

*Nlrx1* “Common Rev”: 5’-CCC AGG CAC TGT TGT CCT ACA-3’

*Nlrx1* “KO For”: 5’-TAA GGG TTC GCG TAC GGT G-3’

### Strains

Two isogenic clones of *Leishmania guyanensis* (*Lgy*) were used. A LRV1-bearing (LRV1^+^ *Lgy*M4147/SSU : IR2SAT-LUC(b)c3) and LRV1-cured (LRV1^-^ *Lgy*M4147/SSU : IR2SAT-LUC(b)c3) *Lgy* (named *Lgy*LRV1+ and *Lgy*LRV1-, respectively) were obtained by drug treatment of LRV1+ strain of *Lgy* M4147 (MHOM/BR/75/M4147) containing a firefly luciferase (ffLUC) gene as described previously (39). *Lgy* parasites were cultured at 26°C in Schneider’s Drosophila medium (Gibco) supplemented with 20% of Fetal Bovine Serum (FBS, Gibco), 1% penicillin/streptomycin (BioConcept), 2% HEPES buffer (BioConcept) and 0.6 μg/ml 6-Biopterin (Sigma-Aldrich) and 0.2% Hemin folate (Sigma-Aldrich, Fluka). For infection, parasites were cultured for 6 days to obtain stationary phase infectious metacyclic promastigotes.

### Bone Marrow Derived Macrophage (BMDM) Culture, Infection and Stimulation

BMDMs were isolated from tibias and femurs of non-infected female and male WT and *Nlrx1* ^-/-^ mice. Macrophages were cultured at 37°C and 5% CO_2_ in complete Dulbecco’s modified Eagle Medium (DMEM) supplemented with 10% FBS, 1% penicillin/streptomycin, 1% HEPES buffer and 50 ng/ml of murine recombinant mouse macrophage colony stimulating factor (rmM-CSF, Immunotools) for 6 days. At day 3, fresh complete DMEM supplemented with rm-MCSF was added. At day 6, adherent BMDMs were isolated and plated at a concentration of 1.25 x 10^6^ cells/ml one day prior of infection. BMDMs were infected at 35°C and 5% CO_2_ with stationary phase parasites with a multiplicity of infection (MOI) of 5 parasites per macrophage or stimulated with 2 μg/ml of polyinosinic-polycytidylic acid (poly I:C) (Immunotools).

### Mice Infection and Quantification of Inflammation and Parasite Burden by Bioluminescence

Age-matched (6-9 weeks old) female or male mice were injected in the hind footpads with 3 x 10^6^ stationary phase *Lgy* promastigotes in 50 μl of Dulbecco’s Phosphate-Buffered Saline (dPBS, Gibco). To follow disease progression, footpad swelling was measured weekly using a Vernier caliper. When required, to quantify inflammation and parasite burden, mice were injected intraperitoneally (i.p.) with 200 mg/kg of Luminol sodium salt (Carbosynth) or 150 mg/kg of VivoGlo Luciferin (Promega) diluted in dPBS, respectively. Bioluminescence from mouse footpads was measured by *In-vivo* Xtreme II (BRUKER) and quantified using Molecular Imaging (MI) software (BRUKER) as described previously (40).

### Histology and Immunohistochemistry (IHC)

Footpads were collected and fixed overnight at 4°C with 4% paraformaldehyde (PFA, Fluka). Following fixation, tissue samples were dehydrated and included in paraffin. 3.5 μm paraffin sections were generated using a Microm HM355 microtome (Thermo Scientific) and stained with hematoxylin (J.T Baker) and eosin (Merck) stain. Sections were visualized using a NanoZoomer S60 (Hamamatsu Photonics K.K.) scanner with Nikon Plan Apochromat 40x objective using brightfield contrast and analyzed using NPD.scan3.3 (Hamamatsu Photonics K.K.). Representative images of the sections are shown.

### RNA Extraction From Footpads and qRT-PCR

Footpads from infected WT or *Nlrx1^-/-^* mice were collected and snap-frozen in liquid nitrogen and kept at -80°C for storage. For RNA extraction, tissues were lysed in TRI Reagent (Molecular Research Center, inc) using a TissueLyser system (Qiagen). RNA was isolated by chloroform/isopropanol/ethanol phase separation protocol as described previously (41). RNA was quantified using NanoDrop 2000 (ThermoFisher Scientific) and 2000 ng/μl of RNA was used for cDNA synthesis. Alternatively, BMDMs were lysed with PRImeZOL Reagent (Canvax) and RNA was isolated using Direct-zol-96 RNA (Zymo Research) according to the manufacturer’s instructions. cDNA was synthesized using SuperScript II Reverse Transcriptase (Invitrogen). Real-time quantitative PCR (qRT-PCR) was performed using the LightCycler 480 (Roche). The results were analyzed using the threshold cycle (C_T_) method (2^-ΔΔCt^) for relative quantification of gene expression and normalized to *L32* housekeeping gene encoding for 60S ribosomal protein. The oligonucleotides used were:

*L32:* 5′-AAG CGA AAC TGG CGG AAA C-3’ and 5′-TAA CCG ATG TTG GGC ATC AG-3’

*Il6*: 5’-TCC AGT TGC CTT CTT GGG AC-3’ and 5’-GTC TAA TTA AGC CTC CGA CT-3’

*Tnfa:* 5’-CAT CTT CTC AAA ATT CGA GTG ACA A-3’ and 5’- TGG GAG TAG ACA AGG TAC AAC CC-3’

*Ifnb:* 5’-AAC CTC ACC TAC AGG GC-3’ and 5′-CAT TCT GGA GCA TCT CTT GG-3’

*Nlrx1:* 5’-CAT GGA AAC TCG GCA GAC AG-3’ and 5’-GGC TAA ACC ACT CGG TGA GG-3’

### Western Blot Analysis

BMDMs were lysed with 1.5x Laemmli’s Sample Buffer in H_2_O and incubated at 95°C for 3 min. Cell lysates were size-fractioned by 8% SDS-PAGE and wet-transferred to a nitrocellulose membrane. Membranes were blocked with 5% non-fat dry milk in Tris buffered saline with 0.1% Tween-20 (TBST) at room temperature. Western blotting was performed using the following antibodies: anti-NLRX1 (1/1000, Proteintech, 17215-1-AP), anti-y-TUBULIN (1/10 000, Sigma-Aldrich, T5326), goat anti-rabbit IgG (H+L) HRP (1/2500, Promega, W4011) and goat anti-mouse IgG (H+L) HRP (1/2500, Promega, W4021). ECL Western Blotting detection reagent (GE Healthcare Life Sciences) was used for revelation.

### Enzyme-Linked Immuno-Sorbent Assay (ELISA)

The concentrations of IFNβ (Thermo Fisher, 424001), IL-6 (Invitrogen, 88-7064-88) and TNF-α (Invitrogen, 88-7324-88) in collected cell-free supernatants at 24 hours from infected or stimulated BMDMs were determined by ELISA following the manufacturer’s instructions. Optical density was read on a Synergy HT Multi-Mode Plate Reader (BioTek Instruments) at 450/570 nm.

### High Throughput Microscopy

BMDMs were seeded in μ-Plate 96 Well Black (Ibidi) at a concentration of 1.25 x 10^6^ cells/ml. Cells were infected or stimulated for 8 and 24 hours and fixed with 4% PFA (Fluka). Cells were subsequently stained with 4′,6-diamidino-2-phenylindole (DAPI) (Molecular Probes) and Alexa Fluor 488 phalloidin (Molecular probes) to stain the nuclei and cytoplasm, respectively. Images were acquired using ImageXpress Micro Confocal (Molecular Devices) with a 40x objective. Parasite and cell number per well were quantified using MetaExpress custom Module Editor (Molecular Devices) as described previously (42).

### RNA Sequencing of BMDMs and Bioinformatics Analysis

BMDMs from age-matched WT and *Nlrx1^-/-^* mice were infected with *Lgy* parasites or stimulated with poly I:C for 8 and 24 hours. RNA was extracted using a RNeasy Kit (Qiagen) following manufacturer’s instructions. RNA quality and concentrations were determined by Fragment Analyzer and Ribogreen QubIT quantification, respectively, and libraries for sequencing were then prepared at the Lausanne Genomic Technologies Facility (GTF). Statistical analysis was performed for genes independently in R (R version 4.0.3). Genes with low counts were filtered out according to the rule of 1 count(s) per million (cpm) in at least 1 sample. Differential expression was computed with limma by fitting data to a linear model (43). Weighted gene co-expression network analysis (WGCNA) was performed on normalized data in R (package WGCNA 1.69). Modules were identified by dynamic tree cut with a minimum module size=20. Module eigengenes (MEs) that are the first principal component of the module were calculated and relationship of module eigengenes with infection status was assessed with a regression analysis. Module eigengenes average predictions were plotted as a heatmap. Gene Ontology (GO) enrichment analysis was performed for gene co-expression modules against GO categories using the topGO R package (topGO 2.26.0) and gene ontology database (07.2019). Only biological processes (BP) were considered for the analysis. For all modules, genes were ranked according to their connectivity within a single module as measured by the kwithin-index.

### Metabolism Assessment by Seahorse Analyzer

Metabolism measurement was performed with a Seahorse XFe96 Extracellular Flux Analyzer (Agilent). BMDMs were plated in a Seahorse XF96 cell culture microplate (Agilent) overnight. Cells were then infected with *Lgy* parasites for 8 hours and then pre-incubated in assay medium (Seahorse XF DMEM pH 7.4, Agilent) supplemented with 2 mM L-glutamine (Gibco), 1 mM pyruvate (Gibco) and 25 mM glucose (Gibco) for 1h at 37°C without CO_2_. To measure mitochondrial metabolism and glycolysis, a Mito Stress Test and a Glycolytic Rate Assay were performed, respectively, according to manufacturer’s instructions. Cells were treated with 1 μM Oligomycin (Sigma), 2 μM FCCP (Sigma), 0.5 μM/0.5 μM Rotenone/Antimycin A (Sigma) and 50 mM 2-deoxy-D-glucose (Sigma). Results were analyzed using Wave Desktop software (Agilent) and data was normalized to total protein concentration per well. Briefly, post-assay, cells were lysed with a mixture of RIPA Buffer IV (Biotech) and a complete protease inhibitor cocktail tablet (Roche) in H_2_O. Protein concentration was quantified using Pierce BCA Protein Assay Kit (Thermo Fisher Scientific) following manufacturer’s instructions. To assess the effect of estradiol, cells were pre-treated with 200 pg/ml of 17-β estradiol (Sigma) for 2 hours and estradiol was kept in the assay medium for the duration of the assay.

### Measurement of ROS Production

Intracellular and mitochondrial ROS production were measured by the superoxide indicator dihydroethidium (DHE) (Thermo Fisher Scientific) and the mitochondrial superoxide indicator MitoSOX Red (Thermo Fisher Scientific). Briefly, cells were infected with *Lgy* parasites or treated with poly I:C for 8 hours, after which cells were labelled with 5μM DHE or 5 μM MitoSOX Red in PBS with 5 mM glucose (Gibco) for 20 min at 37°C. After incubation, cells were washed, and fluorescence was measured at 518/606 nm (MitoSOX Red) and 510/590 nm (DHE) using the Spectramax i3 plate reader (Molecular Devices). Measures were normalized to total protein concentration per well using Pierce BCA Protein Assay Kit (Thermo Fisher Scientific) following manufacturer’s instructions.

### Electron Microscopy Analysis of Mitochondria

After 8 hours of infection, cells were fixed in 2.5% glutaraldehyde solution (Fluka) in PBS for 1 hour at room temperature (RT), then postfixed with a mixture of 1% osmium tetroxide (EMS) and 1.5% of potassium ferrocyanide (Sigma) in PBS for 1 hour at RT. Samples were washed in distilled water, spin down in low melting 2% agarose (Sigma) in H_2_O (Sigma), let to solidify on ice, cut in 1 mm^3^ cube and dehydrated in acetone solution (Sigma) at graded concentrations (30%, 40 min; 50%, 40 min; 70%, 40 min; 100%, 2x1 hour). This was followed by infiltration in Epon (Sigma) at graded concentrations (Epon 1/3 acetone, 2 hours; Epon 3/1 acetone, 2 hours; Epon 1/1, 4 hours; Epon 1/1, 12 hours) and finally polymerized for 48 hours at 60°C. Ultrathin sections of 50 nm were cut on a Leica Ultracut (Leica Mikrosysteme GmbH) and picked up on a copper slot grid 2x1 mm (EMS) coated with a polystyrene film (Sigma). Sections were poststained with 2% uranyl acetate (Sigma) in H_2_O for 10 minutes, rinsed several times with H_2_O followed by Reynold’s lead citrate in H_2_O (Sigma) for 10 minutes and rinsed several times with H_2_O. Singles micrographs were taken with a transmission electron microscope Philips CM100 (Thermo Fisher Scientific) at an acceleration voltage of 80kV with a TVIPS TemCam-F416 digital camera (TVIPS GmbH). To determine the percentage of mitochondria volume per cell volume, a grid (500 nm spacing) was applied on each micrograph and each intersection was defined as being part of the mitochondria, nucleus, or cytoplasm. The stereology analysis was performed using 3dmod and its stereology plugin (44).

### Statistical Analysis

All graphs and statistical tests were generated in GraphPad Prism [version 9.3.1 (350)]. Either unpaired Student’s t-test or two-way ANOVA with multiple comparisons was used for bar graphs, while repeated-measure two-way ANOVA with Bonferroni’s post-test correction was used for x/y curves. Significance was reached with p values ≤ 0.05. p values are shown as * for p < 0.05, ** for p < 0.01, *** for p < 0.001 and **** for p < 0.0001.

## Results

### Loss of NLRX1 Exacerbated Inflammation and Tissue Damage Following *Lgy*LRV1+ Infection of Female Mice

**Figure 1:**
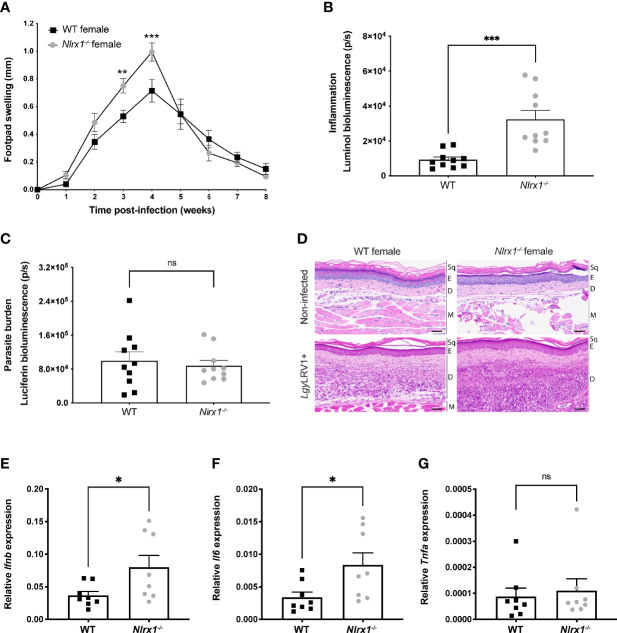
NLRX1 attenuated inflammation and tissue damage in infected female mice. Wild-type (WT) and *Nlrx1*-deficient (*Nlrx1^-/-^*) C57BL/6 female mice (n=5 mice per group) were infected in both hind footpads with 3x10^6^ stationary phase *Lgy*LRV1+ parasites containing a luciferase gene. **(A)** Footpad swelling was measured weekly as a proxy of disease progression. At the peak of infection [4 weeks post-infection (p.i.)] **(B)** *in vivo* inflammation and **(C)** parasite burden was visualized and quantified by bioluminescence imaging after luminol and luciferin injection, respectively. Graphs are presented as mean +- SEM and are representative of three independent experiments. **(D)** Representative images of hematoxylin and eosin (H&E) staining of footpad sections. Upper panel show normal histological appearance of the epidermis and dermis of female WT and *Nlrx1^-/-^* mouse footpads. Bottom panel: lesions from hind footpads of *Lgy*LRV1+ infected female mice were dissected at 4 weeks p.i. and cell recruitment to lesion site was visualized. Magnification: 40x, scale bar: 50 μm. Sq, squames. E, epidermis. D, dermis. M, muscle. (n=5 mice per group). Relative mRNA levels of pro-inflammatory genes **(E)** *Ifnb*, **(F)** *Il6* and **(G)** *Tnfa* were quantified in the lesions of infected WT and *Nlrx1^-/-^* female mice at 4 weeks p.i. using RT-qPCR. Graphs are presented as mean +- SEM (n=8 mice per group). Statistical significance was assessed by two-way ANOVA with multiple comparisons **(A)** or unpaired, parametric t-test (B-C, E-G). ns = non-significant, *p ≤ 0.05 **p ≤ 0.01, ***p ≤ 0.001.

To investigate whether NLRX1 modulated the pathogenicity of *Lgy* and affected disease progression, we first infected female C57BL/6 wild-type (WT) or NLRX1-deficient (*Nlrx1^-/-^*) mice with *Lgy* parasites containing the dsRNA LRV1 virus (*Lgy*LRV1+) and monitored lesion development weekly. In comparison to WT female mice, *Nlrx1^-/-^* infected female mice showed significantly increased footpad swelling (**Figure 1A**) and signs of inflammation as measured by *in vivo* bioluminescence imaging following luminol injection (**Figure 1B**). However, no significant differences were observed in parasite load as measured by bioluminescence of luciferase expressing parasites (**Figure 1C**). Thus, in female mice, NLRX1 seemed to attenuate LRV1 mediated inflammation independently of the parasite load.

To further confirm the role of NLRX1 in regulation of inflammation, we collected non-infected and infected footpads at the peak of infection. We did not observe any differences or abnormalities in skin structure of non-infected *Nlrx1-*deficient mice compared to WT (**Figure 1D**, upper panel). However, correlating to the lesion severity, we observed an important increase in thickness and in immune cell infiltration in the dermis (“D”) of *Lgy*LRV1+ infected *Nlrx1* ^-/-^ mice (**Figure 1D**, bottom panel) compared to WT mice. In addition, we observed that at the peak of infection pro-inflammatory markers *Ifnb* and *Il6* (**Figures 1E, F** **)** were significantly upregulated in lesions of *Nlrx1^-/-^* mice infected with *Lgy*LRV1+ parasites, while no differences were observed in *Tnfa* expression as measured by qRT-PCR (**Figure 1G**). Thus, taken together these results supported a role for NLRX1 in controlling inflammation by limiting immune cell infiltration and tissue damage as well as type I IFN and IL-6 expression in lesions of female mice, while not affecting TNFα expression known to be responsible for parasite killing (36).

### *In Vitro* Analysis of Female BMDMs Suggested a Role for NLRX1 in the Regulation of Inflammation, Infection, Metabolism, and Sex Hormone Signaling

**Figure 2:**
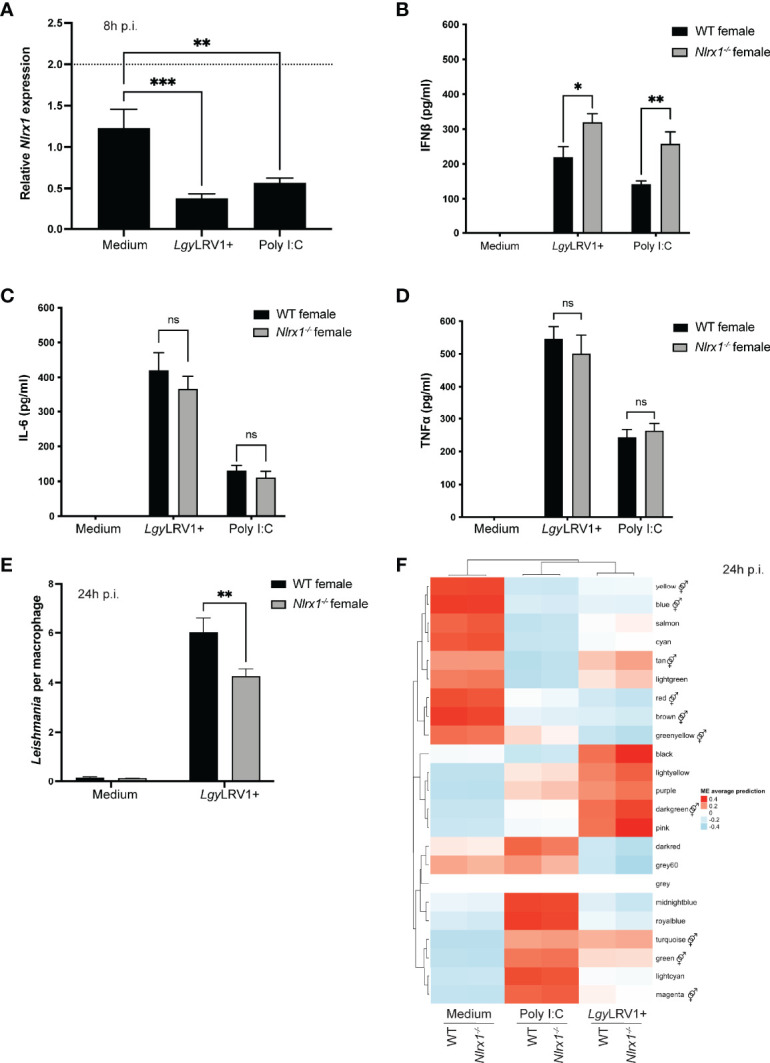
Inflammatory profile and transcriptomics analysis of female BMDMs. Bone marrow derived macrophages (BMDMs) from WT and *Nlrx1^-/-^* female mice were isolated and infected with stationary phase *Lgy*LRV1+ parasites or stimulated with the TLR3 agonist poly I:C (2 μg/ml). *Nlrx1* mRNA levels were quantified by qRT-PCR **(A)** at 8 hours p.i. (n=3 independent experiments). **(B–D)** After 24 hours, supernatants were collected and IFNβ, IL-6 and TNFα secretion was quantified by ELISA in *Lgy*LRV1+ or poly I:C stimulated BMDMs. (n=3 independent experiments). **(E)** At 24 hours p.i., BMDMs were fixed with 4% PFA and stained with DAPI and phalloidin. Cells were visualized with a high content microscope (40x) and intracellular parasite load was quantified using a MetaXpress software (n=2 independent experiments). **(F)** Transcriptomics analysis of WT and *Nlrx1^-/-^* female BMDMs (n=3 mice per group) infected with *Lgy*LRV1+ parasites or stimulated with poly I:C (2 μg/ml) for 24 hours. The heatmap represents the global weighted correlation network analysis (WGCNA) and module names are represented by a color. A gene ontology (GO) enrichment analysis for each module was performed to identify the biological processes associated to each module. 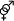 represents modules enriched in GO terms associated with sex hormone signaling. Graphs are presented as mean +- SEM and significance was tested by two-way ANOVA with multiple comparisons (A-F). ns = non-significant, *p ≤ 0.05, **p ≤ 0.01, ***p ≤ 0.001.

Several studies have reported a downregulation of NLRX1 in different experimental models such as viral and bacterial infections and brain injury (27, 45, 46). Therefore, we investigated whether infection with *Lgy* regulated *Nlrx1* expression. Thus, we infected bone marrow derived macrophages (BMDMs) isolated from WT female mice with *Lgy*LRV1+ parasites or stimulated them with polyinosinic-polycytidylic acid (poly I:C), a known synthetic dsRNA agonist of TLR3. We observed a downregulation of *Nlrx1* mRNA in both infected and poly I:C treated BMDMs at 8 hours post-infection (p.i.) when infection is established, however with a fold-change inferior to 2 (**Figure 2A**). This downregulation was no longer observed at 24 hours p.i. (**Supplementary Figure S1A** **)**. Similarly, we did not observe any significant changes in NLRX1 protein levels at 8 or 24 hours p.i. (**Supplementary Figure S1B**), suggesting a transcriptional regulation of NLRX1 only in the early phase of infection with *Lgy*LRV1+ parasites or after poly I:C treatment.

To further characterize the increased tissue damage and inflammation observed in *Nlrx1^-/-^* female mice, we next sought to determine any possible effect of NLRX1-deficiency on inflammation and *Lgy* infection in *in vitro* infected BMDMs. Thus, we infected WT and *Nlrx1* ^-/-^ female BMDMs with *Lgy*LRV1+ or stimulated them with poly I:C and measured pro-inflammatory cytokines in the cell-free supernatant at 24 hours post-infection. As expected, no cytokines were detected in non-infected cells. In contrast, NLRX1-deficiency resulted in a significantly increased production of IFNβ in *Lgy*LRV1+ infected or poly I:C treated cells (**Figure 2B**). We did not observe any differences in IL-6 or TNFα production (**Figures 2C, D** **)** suggesting that IFNβ was produced by infected macrophages whereas, upon tissue damage, *Il6* up-regulation measured in lesions was likely produced by other sources such as keratinocytes, dendritic cells and fibroblasts (47).

In infectious models, loss of NLRX1 has been reported to promote either pathogen survival or clearance depending on the model (46, 48–52). To investigate whether NLRX1 affected the number of parasites per BMDM, we infected WT and *Nlrx1*-/- female BMDMs with *Lgy*LRV1+ parasites for 8 and 24 hours. We did not observe differences in parasite burden in the establishment of infection (8 hours p.i.) (**Supplementary Figure S1C**) suggesting that NLRX1 did not affect the phagocytic capacity of BMDMs. In contrast, at 24 hours post infection, the absence of NLRX1 resulted in a decreased number of parasites per cell (**Figure 2E**). This decrease was associated to an increased macrophage survival in absence of NLRX1 that was observed only at 24 hours p.i. (**Supplementary Figures S1D, E**) (27, 53). However, as shown in **Figures 1A, C**, despite an increased macrophage survival with a lower number of parasites per cell *in vitro, in vivo Nlrx1-*deficient female mice developed larger lesions independently of the parasite load.

To better define the role of this mitochondrial sensor in macrophages isolated from female mice, we performed a transcriptomics analysis of WT and *Nlrx1^-/-^* female BMDMs infected with *Lgy*LRV1+ parasites or stimulated with poly I:C for 8 or 24 hours. We then performed a global weighted correlation network analysis (WGCNA) to group genes with similar expression patterns into modules. WGCNA has been used previously to identify key biological processes and gene modules associated with the studied disease (54–57). The underlying hypothesis is that genes involved in the same function or pathway or that are co-regulated are expected to be in the same module named by a color. Genes that do not group to any module form the “grey” module and are discarded from the analysis. The association of the WGCNA modules with the different conditions is represented as heatmaps at 8 and 24 hours (**Figure 2F**, **Supplementary Figure S1F**). The genes in each module are listed in **Supplementary Tables 1, 2**. We performed a gene ontology (GO) enrichment analysis for each module to identify the biological processes associated to each module. For each module GO terms are listed in **Supplementary Tables 3 and 4**. Based on the GO analysis, we could group most of the modules into 2 main categories of GO terms (1): inflammation and infection and (2) mitochondria and metabolism (**Supplementary Figure S1G**). These categories were based on a selection of several keywords such as “interleukin” or “biosynthesis”, respectively. Identification of such biological processes was not surprising because of the choice of an infectious model and a knock-out gene for a mitochondrial receptor described to play a role in both categories. In contrast, both at 8 and 24 hours p.i., more than a third of the modules (34.8% and 45.5%, respectively) were enriched in GO terms associated to sex hormone signaling (**Figure 2F**, **Supplementary Figures S1F, G**) such as “regulation of androgen receptor signaling pathway” (GO:0060765) or “cellular response to estrogen stimulus” (GO:0071391). These GO terms were rather unexpected and suggested a potential sex-bias in the NLRX1 phenotype.

### In Male Mice Loss of NLRX1 Did Not Affect Lesion Severity

**Figure 3:**
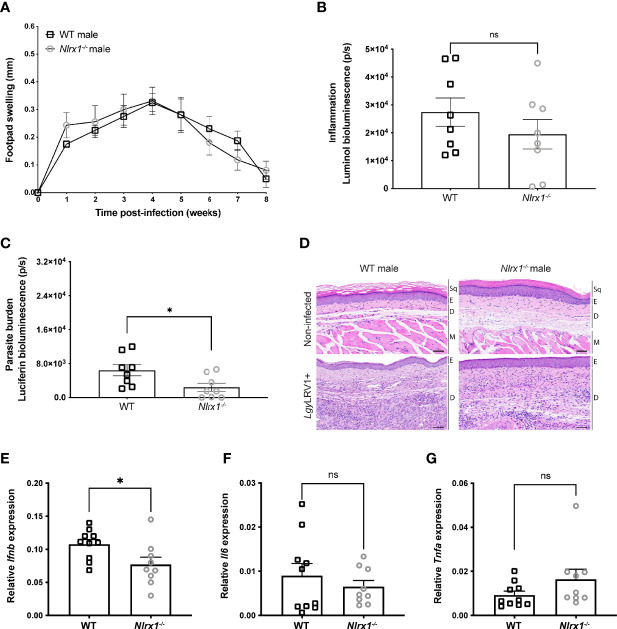
In male mice NLRX1 did not regulate inflammation or tissue damage. Wild-type (WT) and *Nlrx1*-deficient (*Nlrx1^-/-^*) C57BL/6 male mice (n=4 mice per group) were infected in both hind footpads with 3x10^6^ stationary phase *Lgy*LRV1+ parasites **(A)** Footpad swelling was measured weekly as a proxy of disease progression. At the peak of infection (4 weeks p.i.), **(B)** *in vivo* inflammation and **(C)** parasite burden was visualized and quantified by bioluminescence imaging after luminol and luciferin injection, respectively. Graphs are presented as mean +- SEM and are representative of three independent experiments. **(D)** Representative images of hematoxylin and eosin (H&E) staining of footpad sections. Upper panel show normal histological appearance of the epidermis and dermis of male mouse footpads. Bottom panel: lesions from hind footpads of *Lgy*LRV1+ infected male mice were dissected at 4 weeks p.i. and cell recruitment to lesion site was visualized. Magnification: 40x, scale bar: 50 μm. Sq, squames. E, epidermis. D, dermis. M, muscle. (n=5 mice per group). Relative mRNA levels of pro-inflammatory genes **(E)** *Ifnb*, **(F)** *Il6* and **(G)** *Tnfa* were quantified in the lesions of infected WT and *Nlrx1^-/-^* male mice at 4 weeks p.i. using RT-qPCR. Graphs are presented as mean +- SEM. (n=9-10 mice per group). Statistical significance was assessed by two-way ANOVA with multiple comparisons **(A)** or unpaired, parametric t-test **(B, C, E–G)**. ns = non-significant, *p ≤ 0.05.

Although no association between NLRX1 and sex hormone signaling has been described to our knowledge, many studies have shown that sex hormones may play a role in the regulation of innate immune cell activation, inflammation, and mitochondrial function (1, 58). Based on the transcriptomics analysis of female BMDMs, we decided to investigate whether the *Nlrx1^-/-^* phenotype was dependent on sex. Thus, we infected male C57BL/6 WT or *Nlrx1^-/-^* mice with *Lgy*LRV1+ parasites. In contrast to female mice, we found no significant differences in the development of lesions (**Figure 3A**). Moreover, at the peak of infection, male mice did not display any differences in inflammation as measured by *in vivo* bioluminescence imaging (**Figure 3B**). However, we observed a reduced parasite burden in *Nlrx1^-/^-* mice in comparison to WT (**Figure 3C**) that we did not observe in female mice (**Figure 1C**). Consistent with the absence of difference in inflammation and contrary to female mice, we observed no significant differences in skin structure, dermal thickness or immune cell infiltration between non-infected (**Figure 3D**, upper panel) or *Lgy*LRV1+ infected male mice (**Figure 3D**, bottom panel). Finally, we observed a significant reduction in *Ifnb* mRNA levels in the lesions of *Nlrx1^-/-^* male mice infected with *Lgy*LRV1+ (**Figure 3E**) in comparison to WT, but no differences in *Il6* or *Tnfa* mRNA levels as measured by qRT-PCR (**Figures 3F, G** **)**. Taken together, NLRX1 did not modulate lesion development or inflammation in male mice contrarily to results obtained with female mice suggesting a strong sex bias in the function of NLRX1.

### Female and Male BMDMs Showed Differences in Inflammation and Infectivity in Absence of NLRX1

**Figure 4:**
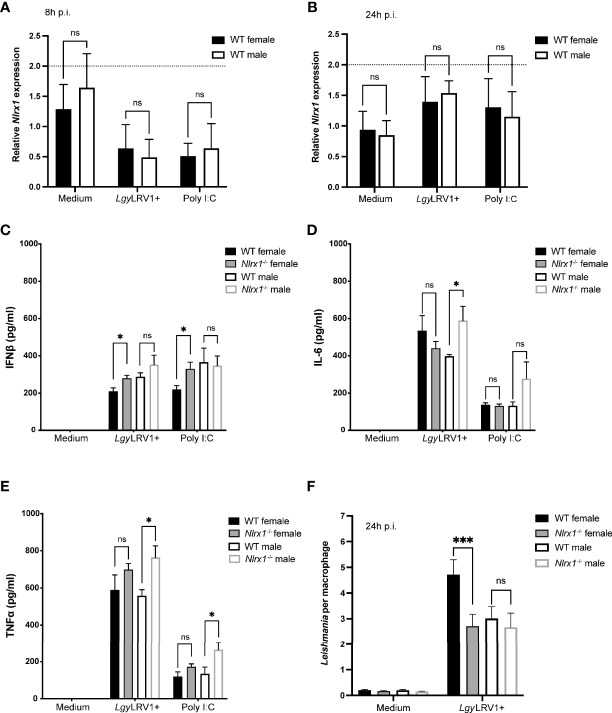
Sex bias in inflammation and infection in absence of NLRX1. BMDMs from female and male WT and *Nlrx1^-/-^* mice were isolated simultaneously and infected with *Lgy*LRV1+ parasites or stimulated with TLR3 agonist poly I:C (2 μg/ml). After 8 hours **(A)** and 24 hours p.i. **(B)**, *Nlrx1* mRNA levels were quantified by qRT-PCR (n=3 independent experiments). **(C–E)** After 24 hours, proinflammatory cytokines IFNβ, IL-6 and TNFα were quantified in cell-free supernatants by ELISA in *Lgy*LRV1+ infected or poly I:C stimulated BMDMs. (n=3-4 independent experiments). **(F)** At 24 hours p.i., BMDMs were fixed with 4% PFA and stained with DAPI and phalloidin. Cells were visualized with a high content microscope (40x) and intracellular parasite load was quantified using a MetaXpress software. (n=3 independent experiments). Graphs are presented as mean +- SEM and significance was tested by two-way ANOVA with multiple comparisons **(A–F)**. ns = non-significant, *p ≤ 0.05, ***p ≤ 0.001.

To better understand the sex-bias in NLRX1 function, we decided to investigate whether isolated macrophages also displayed a sex-dependent phenotype. First, we infected both female and male WT BMDMs with *Lgy* parasites or stimulated them with poly I:C for 8 and 24 hours to analyze whether NLRX1 expression was affected by sex. At 8 or 24 hours p.i., we did not observe any significant differences in *Nlrx1* mRNA (**Figures 4A, B** **)** and protein levels (**Supplementary Figure S2A**) between females and males, suggesting that biological sex did not directly affect NLRX1 expression in BMDMs. Based on the *in vivo* data, the role of NLRX1 in the regulation of inflammation and infection was highly dependent on sex. Thus, we measured IFNβ, IL-6 and TNFα cytokine production at 24 hours post-infection in both female and male WT and *Nlrx1^-/-^* BMDMs infected with *Lgy*LRV1+ parasites or stimulated with poly I:C. No cytokines were detected in non-infected cells. Consistent with the lesions, increased IFNβ production (**Figure 4C**; **Supplementary Figure S3A**) was specific to female *Nlrx1^-/-^* BMDMs infected with *Lgy*LRV1+ or treated with poly I:C, whereas we did not observe any differences between male BMDMs. Interestingly, the Nlrx1^-/-^ female BMDMs showed a similar IFNβ production compared to males. Surprisingly and in contrast to the lesions, NLRX1-deficiency resulted in a significantly increased production of both IL-6 and TNFα levels (**Figures 4D, E**; **Supplementary Figures S2B, S3B, C**) in *Nlrx1^-/-^* male BMDMs in comparison to WT male BMDMs.

Since we observed differences in cytokine profiles of female and male BMDMs, we investigated whether also parasite burden in BMDMs was affected by sex. As previously, we infected WT and *Nlrx1* ^-/-^ female and male BMDMs with *Lgy*LRV1+ parasites for 8 and 24 hours. At 8 hours p.i. female and male BMDMs showed similar parasite burdens independently of the genotype (**Supplementary Figure S2C**). However, at 24 hours p.i. WT female BMDMs maintained a higher parasite burden, whereas female *Nlrx1^-/-^* BMDMs and both male BMDMs showed a significantly reduced and similar parasite burden (**Figure 4F**; **Supplementary Figure S3D**). Although we did not observe statistically significant differences in macrophage survival, at 24 hours but not at 8 hours p.i., both male BMDMs and *Nlrx1-*deficient female BMDMs seemed to survive better in comparison to WT female cells correlating to the lower parasite burden observed in these cells (**Figure S2D, E**). Taken together, these results supported a strong association between sex and NLRX1 function.

### Both Sex and Genotype Affected Metabolic Profiles and Mitochondria of BMDMs

**Figure 5:**
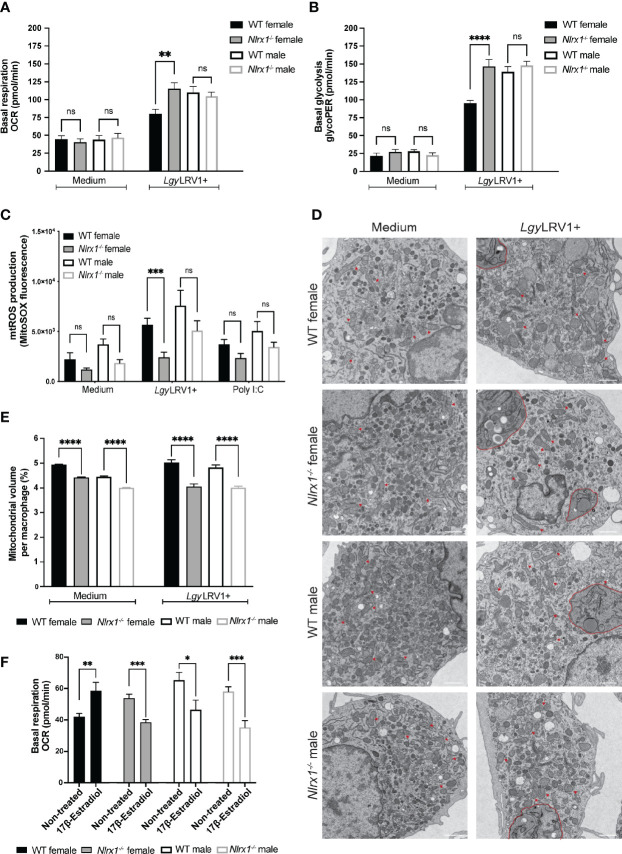
In absence of NLRX1 female BMDMs had a male-like metabolic response. BMDMs from female and male WT and *Nlrx1^-/-^* mice were isolated simultaneously and infected with *Lgy*LRV1+ parasites for 8 hours. After 8 hours, **(A)** basal mitochondrial respiration and **(B)** basal glycolytic rate were assessed by Seahorse XFe96 analyzer and adjusted to protein concentration per well (n=3-4 independent experiments). **(C)** Mitochondrial ROS (mtROS) production was quantified in female and male BMDMs infected with *Lgy*LRV1+ parasites or treated with poly I:C (2 μg/ml) for 8 hours. Cells were stained with MitoSOX Red (5 μM) for 20 min at 37°C. Fluorescence was measured using a Spectramax i3 plate reader and adjusted to protein concentration per well. (n=3 independent experiments). **(D)** Mitochondria structure and **(E)** the percentage of mitochondria volume per cell of female and male BMDMs infected with *Lgy*LRV1+ parasites for 8 hours were analyzed by transmission electron microscopy. Representative images are shown. Red arrows show examples of normal mitochondrial structure. *Lgy*LRV1+ parasites are contoured in red (n=2 independent experiments, total of minimum 60 cells analyzed per group). Magnification: 4800x. Scale bar: 1 μm. **(F)** BMDMs from female and male WT and *Nlrx1^-/-^* mice were pre-treated with 17β-estradiol (200 pg/ml) for 2 hours and estradiol was kept in the assay medium for the duration of the assay. Basal mitochondrial respiration was assessed by Seahorse XFe96 analyzer and adjusted to protein concentration per well (n=4 independent experiments). Graphs are presented as mean +- SEM and significance was assessed by two-way ANOVA with multiple comparisons **(A–C)** or unpaired, parametric t-test **(E, F)**. ns = non-significant, *p ≤ 0.05, **p ≤ 0.01, ***p ≤ 0.001, ****p ≤ 0.0001.

NLRX1 was shown to play a role in the regulation of both OXPHOS and glycolysis, the two major metabolic pathways for energy production (31, 59). In addition, based on the transcriptomics analysis of female BMDMs, NLRX1 seemed to have a strong effect on cellular metabolism and mitochondrial function. Thus, we sought to investigate whether the loss of NLRX1 resulted in a change in OXPHOS or glycolysis, the two major energetic pathways of the cell. To examine if NLRX1 affected OXPHOS, we determined the oxygen consumption rate (OCR) in both female and male WT and *Nlrx1^-/-^* BMDMs infected with *Lgy*LRV1+ parasites for 8 hours. In non-infected BMDMs, we observed very low OCR levels and no differences between groups, suggesting a low metabolic activity in unstimulated cells after 8 hours (**Figure 5A**). Moreover, NLRX1-deficient female BMDMs infected with *Lgy*LRV1+ showed a significantly increased basal mitochondrial respiration compared to WT female, but that was comparable to basal respiration of male BMDMs. In contrast we did not observe differences in basal respiration between WT and *Nlrx1*-deficient male BMDMs infected with *Lgy*LRV1+ (**Figure 5A**; **Supplementary Figures S4A**, **S5A**). To investigate whether NLRX1 also affected glycolysis in BMDMs, we measured the basal glycolytic rate in the same conditions. In non-infected cells, we observed a very low glycolytic rate, and no differences between groups (**Figure 5B**). Similarly to the pattern observed for mitochondrial respiration, female *Nlrx1^-/-^* BMDMs infected with LgyLRV1+ showed a significantly increased glycolytic rate compared to WT female, but that was similar to the glycolytic rate of male BMDMs. As before for mitochondrial respiration, we did not observe differences in glycolytic rate between WT and *Nlrx1*-deficient male BMDMs (**Figure 5B**; **Supplementary Figure S5B**). Taken together, these results suggested a global effect of NLRX1 on female, but not on male macrophage metabolism.

NLRX1 is localized at the mitochondria, and its function in the control of inflammation has been linked to mitochondria and in the modulation of mtROS production (21, 23, 24, 50, 60, 61). This modulation occurs potentially through the interaction with Ubiquinol-Cytochrome C Reductase Core Protein 2 (UQCRC2), a subunit of the complex III of the respiratory chain (38, 62). We measured mtROS and cellular ROS accumulation, by MitoSOX Red and DHE respectively, in female and male BMDMs infected with *Lgy*LRV1+ parasites or treated with poly I:C for 8 hours. Globally mtROS was reduced in *Nlrx1^-/-^* BMDMs independently of sex, whereas no differences were observed in cellular ROS (**Figure 5C**; **Supplementary Figure S4B, S5C**). To verify whether mitochondrial morphology was affected by NLRX1-defiency, we performed transmission electron microscopy (TEM) on non-infected and *Lgy*LRV1+ infected female and male BMDMs. We did not observe any differences in mitochondrial morphology between WT and *Nlrx1^-/-^* BMDMs and no mitochondrial defects were observed (**Figure 5D**). On the other hand, quantification of TEM images revealed a reduced mitochondrial density and number in both female and male *Nlrx1^-/-^* BMDMs compared to WT (**Figure 5E** **;** **Supplementary Figure S4C, S5D**) potentially explaining the reduced mtROS production observed in these cells.

Since neither mtROS production nor mitochondrial structure could explain differences observed in metabolism, we examined whether sex differences in cellular bioenergetics could be modified by the female hormone 17-β estradiol. Similarly to the study done by Gupta et al. (2020) (3), estradiol treatment of male BMDMs significantly reduced mitochondrial respiration (**Figure 5F**). Surprisingly, estradiol treatment increased basal respiration of WT female BMDMs but reduced basal respiration of *Nlrx1-*deficient female BMDMs similarly to males (**Figure 5F**). This male-like pattern of *Nlrx1-*deficient female cells after estradiol treatment was specific to mitochondrial respiration since estradiol increased the glycolytic rate only in females but not in male BMDMs (**Supplementary Figure S4D**). Taken together, these results suggested that macrophage metabolism can be modulated by the female sex hormone and differences in estradiol response might contribute to the sex bias observed in the bioenergetics between WT and *Nlrx1-*deficient BMDMs.

### In Female Mice NLRX1 Deficiency Resulted in a “Masculinization” of the BMDM Transcriptomic Profile

**Figure 6:**
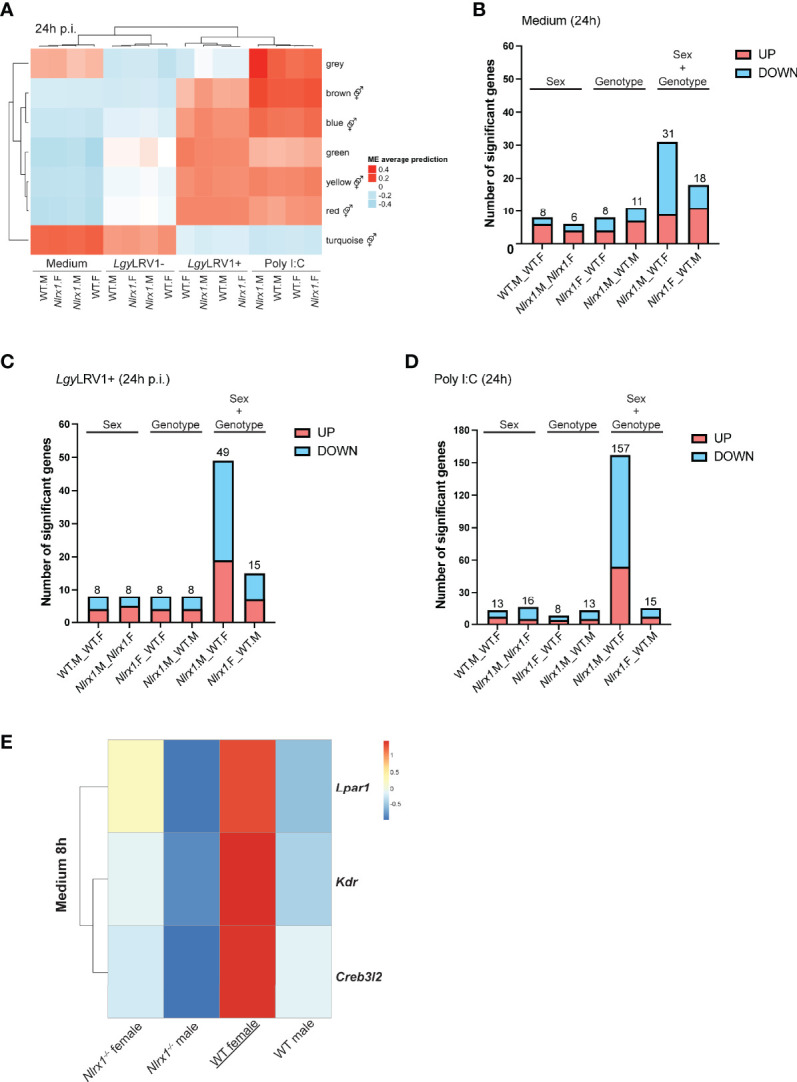
Transcriptomics analysis of female and male BMDMs revealed a male-like phenotype of *Nlrx1^-/-^* female BMDMs. **(A)** Transcriptomics analysis of both female and male WT and *Nlrx1^-/-^* BMDMs (n=3 mice per group) infected with *Lgy*LRV1+ and *Lgy*LRV1- parasites or stimulated with poly I:C (2 μg/ml) for 24 hours. The heatmap represents the global weighted correlation network analysis (WGCNA) and module names are represented by a color. A gene ontology (GO) enrichment analysis for each module was performed to identify the biological processes associated to each module. represents modules enriched in GO terms associated with sex hormone signaling. At 24 hours, the number of differentially expressed genes between groups in **(B)** non-infected, **(C)** *Lgy*LRV1+ infected and **(D)** poly I:C treated conditions is plotted. On each barplot the number represents the total number of differentially upregulated (in red) and downregulated (in blue) genes. Pairwise comparisons are done by “sex” (same genotype, different sex, “WT.F._WT.M” and “*Nlrx1.*F_*Nlrx1.M”)*, by “genotype” (same sex, different genotype (“*Nlrx1.*F_WT.F” and “*Nlrx1.*M_WT.M”), or by combining both “sex and genotype” (“*Nlrx1.*M_WT.F” and “*Nlrx1.*F_WT.M”). See **Supplementary Tables 9–11** for the list of significantly differentially expressed genes. **(E)** Identification of WT female specific genes. Genes that were differentially expressed in only in WT female BMDMs but not between the other three groups (*Nlrx1-/-* female, *Nlrx1-/-* male and WT male BMDMs) were identified in non-infected samples at 8 hours.

To better understand the complex interplay between NLRX1 genotype and sex, we decided to perform a second RNA sequencing and WGCNA analysis including both female and male BMDMs. To further confirm the role of NLRX1 in the control of TLR3- and type I IFN-mediated inflammation, we added a non-inflammatory strain of *Lgy* that does not carry the LRV1 virus (*Lgy*LRV1-) to the analysis. Infection with *Lgy*LRV1- strain does not induce NF-κB mediated pro-inflammatory cytokines nor a potent type I IFN response thus leading to a less severe form of the disease (37, 63). We infected BMDMs with both *Lgy* parasites or stimulated them with poly I:C for 8 or 24 hours. The relationship between the different modules and the experimental conditions was assessed with a regression analysis and module eigengenes average predictions are shown as heatmaps at 24 and 8 hours (**Figure 6A**; **Supplementary Figure S6A**, respectively). The genes in each module are listed in **Supplementary Tables 5, 6**. In each condition, the heatmap clusters the groups according to their similarity. Supporting the male-like behavior of *Nlrx1-*deficient female cells, we could observe that at 24 hours post-infection, in all condition except with poly I:C treatment, the *Nlrx1^-/-^* female BMDMs clustered more closely to the WT and *Nlrx1-*deficient male BMDMs than to the WT female (**Figure 6A**). At 8 hours p.i., this clusterization pattern was only observed with infection with *Lgy*LRV1+ (**Supplementary Figure S6A**). As previously, we performed a gene ontology (GO) enrichment analysis for each module to identify the biological processes associated to each module. For each module GO terms are listed in **Supplementary Tables 7, 8**. As expected, GO enrichment analysis of the different modules revealed that most modules were enriched in GO terms associated to (1) Inflammation and infectivity and (2) Mitochondria and metabolism. In addition, at 8 hours p.i. more than half of the modules (60%) were enriched in GO terms associated to (3) sex hormone signaling, while at 24 hours p.i. we observed this enrichment in all modules, except the *green* module (**Figure 6A**; **Supplementary Figure S6A, S6B**).

We next performed pairwise comparisons between the different groups and plotted the number of significantly differentially expressed genes both at 8 and 24 hours using a cut-off of fold-change [-2:2] and an adjusted p-value < 0.05 (**Figure 6B–D**; **Supplementary Figures S6C–G**). We identified only a few sex- or genotype-specific genes except in non-infected BMDMs in which at 8 hours p.i. we identified a significant number of genotype-specific genes (**Supplementary Figure S6C**). In the last two comparisons, we compared opposite genotypes with opposite sex. Interestingly, we observed a common pattern that when we compared *Nlrx1^-/-^* female BMDMs to a WT male, independently of the condition or time-point, we observed less differences than when *Nlrx1^-/-^* male BMDMs were compared to a WT female, suggesting that the transcriptomic profile of female *Nlrx1^-/-^* cells was closer to a male, as suggested by the WGCNA analysis.

To investigate whether we would be able to explain why *Nlrx1^-/-^* female BMDMs had a male-like transcriptomics profile, we wanted to identify genes that in *Nlrx1^-/-^* female BMDMs were expressed at a similar level than in male BMDMs. To do so, we looked for genes that were differentially expressed only in the WT female BMDMs, but not between the three other groups, *Nlrx1^-/-^* female, *Nlrx1^-/-^* male and WT male BMDMs. We used a threshold of adjusted p-value inferior to 0.1. The analysis identified only 3 genes in non-infected BMDMs at 8 hours timepoint (**Figure 6E**): the lysophosphatidic acid receptor 1 (*Lpar1*), the vascular endothelial growth receptor factor 2 (*Kdr*) and the cyclic AMP-responsive element-binding protein 3-like protein 2 (*Creb3l2*). These genes play a role in inflammation (64–66), angiogenesis (67) and collagen synthesis (68, 69), respectively, aspects that are hallmarks of *Leishmania* infection (70, 71) and where in other experimental models sex differences have been described (72–74).

## Discussion

It is well established that females and males differ in their clinical manifestations of both infectious and inflammatory diseases due to differences in the development and strength of the immune response (75, 76). In this study, we provided evidence that NLRX1 limited inflammation and tissue damage in female mice infected with the human protozoan parasite *Lgy* carrying an endosymbiotic dsRNA virus, LRV1, activating TLR3. We showed that in presence of this TLR3 agonist, *Nlrx1*-deficient female mice developed a more severe pathology with increased inflammation and immune cell recruitment to the site of infection compared to WT mice independently of the parasite burden measured in the lesions, suggesting a role in the control of inflammation. Here, inflammation in the lesion was mediated by an increased expression of the pro-inflammatory *Il6* and *Ifnb*, but not *Tnfa.* Surprisingly, we did not observe any difference in IL-6 production between *in vitro* female WT and *Nlrx1*-deficient macrophages infected with *Lgy*LRV1+, but only an increased production of IFNβ. Thus, our results suggested that infected macrophages contributed to increased IFNβ, but other cell types in the skin lesion also contributed to increased IL-6 production and to the *in vivo* exacerbated phenotype observed in *Nlrx1*-deficient female mice.

In *Lgy*LRV1+ infection, the exact mechanism of type I IFN regulation by NLRX1 is yet to be further elucidated. Different mechanisms of anti-viral regulation by NLRX1 have been proposed, including inhibition of retinoic acid-inducible gene (RIG)-I-like receptor-mitochondrial antiviral-signaling (RIG-I-MAVS) protein interaction (20, 22), sequestration of the DNA sensor stimulator of interferon genes (STING) (77, 78), competition with the dsRNA activated protein kinase (PKR) (79), or binding to the autophagy complex formed of mitochondrial Tu translation elongation factor and autophagy related proteins 5 and 12 (TUFM/ATG5/ATG12) (52, 80). Several studies support that NLRX1 is located at the mitochondrial matrix rather than at the mitochondrial outer membrane (MOM) as suggested by the first study by Moore *et al.* (20, 62). Therefore, it raises the question how interaction with proteins located on the MOM or in the cytoplasm, such as MAVS or STING, respectively, would occur within the mitochondrial matrix unless disruption of the mitochondria occurs in the experimental model in question. Although we did not observe any mitochondrial structural damage *in vitro* in BMDMs, we cannot exclude that the hypoxic conditions in the lesions in *Lgy* infection would lead to a mitochondrial functional decline as it is often described in injury and ischemia (81–83). However, in a previous study, we have shown that LRV1 does not activate the cytoplasmic RNA sensors such as RIG-I-MAVS signaling and viral RNA recognition occurs only *via* the TLR3 pathway (37, 63, 84). Thus, in our study NLRX1 controlled IFNβ production downstream of TLR3.

To better understand the role of NLRX1 in our model system of infection, we first performed a transcriptomics analysis of infected female BMDMs. Bioinformatic analysis revealed a significant number of modules enriched in GO terms associated to sex hormone signaling. Even if still quite controversial, sex differences in clinical outcome to viral infections have been reported and differences in susceptibility may be attributed to both sex hormones and sex chromosome encoded genes. Although previous reports have not described a link between sex and NLRX1, oppositely to females, infection of *Nlrx1-*deficient male mice did not result in an exacerbated disease outcome. Indeed, male mice did not exhibit any significant differences in lesion size or cell recruitment. Conversely to *in vivo* where *Nlrx1-*deficient male mice showed a slight reduction in *Ifnb* and *Il6* expression, *in vitro Lgy*LRV1+ infected male BMDMs produced higher levels of IL-6 and TNFα, but not IFNβ, in comparison to females, suggesting a role for other inflammatory cells or mediators *in vivo.*

Several PRRs, including TLRs and NLRs, are able to activate multiple metabolic pathways that lead to a metabolic switch from OXPHOS to glycolytic ATP production which is often critical for innate immune cell activation (85, 86). Given its mitochondrial localization, NLRX1 has been suggested to play a role in the maintenance of mitochondrial function and metabolism (31, 81). However, its impact on cellular metabolism seem to depend on the cell type and to our knowledge no studies have investigated the role of NLRX1 on macrophage colony stimulating factor (M-CSF) primed BMDM metabolism (87). *Leishmania* and other intracellular pathogens are known to manipulate host metabolism and *Leishmania* infection seems to favor a switch to aerobic glycolysis (88, 89). Here we showed that infection with *Lgy* parasites induced glycolysis in infected BMDMs while maintaining a high OXPHOS activity. Interestingly, WT male BMDMs showed a higher metabolic activity in comparison to WT females. This is in line with a previous study showing that neutrophils, another innate immune cell type, isolated from males had a higher mitochondrial respiration in comparison to females (3). However, no sex differences in metabolism were observed between female and male *Nlrx1-*deficient BMDMs. Interestingly, the OXPHOS and glycolytic activity of the BMDMs, the parasite burden, the *in vitro* IFNβ production and response to 17β-estradiol all showed a similar pattern: female *Nlrx1-*deficient cells had a profile similar to male cells. Differences in metabolic rates were not linked to mitochondrial numbers or to impairment of mitochondrial structure since *Nlrx1-*deficient cells had less mitochondria and produced less mtROS than WT cells independently of sex and no structural defects were observed upon *Lgy*LRV1+ infection.

Since BMDMs were able to respond to 17β-estradiol, the differences in bioenergetic profiles of male and female BMDMs may be potentially driven by sex hormones. In neutrophils, a higher OXPHOS profile observed in males has been linked to immature state neutrophils (3). How these differences in metabolic profiles impact the macrophage function, should however be further studied. Estrogens have been shown to impact macrophage metabolism directly. Erα-mediated signaling in macrophages have been shown to promote anti-inflammatory M2-polarization and promote wound healing and cutaneous repair by enhancing angiogenesis and collagen synthesis (9, 10, 90–92). Estrogens not only play a role in mitochondrial biogenesis and in the regulation of mitochondrial function, but several studies have shown that estrogens may also play a role in glucose metabolism and may also stimulate glycolysis (93, 94). Reports are still conflicting on the role of NLRX1 on cell metabolism. Therefore, the effect of sex hormones on BMDMs metabolism and on NLRX1 would be of great interest for future studies.

Trying to provide novel insight on the sex-related differences observed in *Nlrx1-*deficient cells, we performed a second transcriptomic analysis of both female and male BMDMs. Interestingly, at 8 hours post-infection, *Nlrx1-*deficient cells clustered together with males with *Lgy*LRV1+ infection, while at 24 hours this clustering occurred in all conditions except treatment with poly I:C. Similarly, pairwise comparisons of significantly differentially expressed genes between groups confirmed a male-like transcriptomic profile of *Nlrx1-/-* female cells. Our analysis identified three WT female signature genes in non-infected BMDMs at 8 hours timepoint: *Lpar1*, *Kdr* and *Creb3l2*. The role and contribution of *Lpar1* to the observed sex differences may be of great interest for future studies. The synthesis of its ligand, lysophosphatidic acid (LPA), was shown to be type I IFN dependent in an autocrine and paracrine manner in response to TLR3 signaling (95). Differential levels of secreted LPA may contribute to differences in inflammation and pathology observed *in vivo* and *in vitro*, since LPA is produced by several cell types. Of note, sex differences in response to LPA have been described in a model of osteoarthritis (96).

NLRX1 is a unique mitochondria-associated innate immune receptor of the NLR family, and its role extends from the traditional pathogen recognition to the regulation of different cellular functions to control inflammation. However, as highlighted by the diverse effects of NLRX1 that have been described, the mechanism through which NLRX1 influences inflammation, the immune response or the metabolism is still under debate. One might speculate that some of the discrepancies observed in the function of NLRX1 may be attributed to the sex of the animal model used in their research. Interestingly, it was shown that the C-terminal leucine-rich repeat domain (LRR) of NLRX1 could bind several polyunsaturated lipids that mediated the anti-inflammatory effects of NLRX1 (31, 97). In serum LPA is most often found in its unsaturated forms and polyunsaturated forms of LPA are synthetized for example in mouse models of allergic airway inflammation (98, 99). Screening of compounds also predicted that other lipids including sterol lipids could modulate NLRX1 activity (97). All sex steroid hormones are derived from cholesterol, the main sterol synthetized in animal cells, and although *Nlrx1* expression did not differ between males and females, it remains to be determined whether sex hormones could bind and modulate NLRX1 function and its downstream signaling.

In addition to cellular and lipid metabolism, infection with *Leishmania* may lead to modifications of the extracellular matrix (ECM) and collagen composition of the dermis at the site of infection (70, 100). In addition, cutaneous leishmaniasis is also characterized by vascular remodeling and lymphangiogenesis mediated by the vascular endothelial growth factor A (VEGF-A)/VEGF receptor 2 (VEGFR-2) signaling pathway that is essential for lesion resolution (71, 101, 102). Interestingly, in addition the *Lpar1*, two other gene candidates were identified in our transcriptomic analysis to be upregulated only in WT females, *Creb3l2* and *Kdr* that play a role in collagen synthesis (68) and angiogenesis (67), respectively. NLRX1 has been shown to affect both. Overexpression of NLRX1 human nucleus pulposus cells in the intervertebral disc resulted in increased collagen synthesis and decreased ECM decomposing enzymes (103). On the other hand, *Nlrx1-*deficiency led to increased expression of wound healing factors epidermal growth factor (EGF) and TGFβ in epithelial cells in a mouse model of DSS-induced colitis (104). Thus, both angiogenesis and collagen composition could be further investigated in infected footpad sections of both female and male wild-type and *Nlrx1-*deficient mice.

Taken together, our study provides novel insight on the relevance of the first mitochondrial NLR and its connection to the control of inflammation specifically in females. There is accumulating evidence that in human diseases innate immune response, inflammation, and energy metabolism are regulated in a sex-dependent manner (105, 106). Increasing number of studies have shown altered expression of NLRX1 in human patients. For example, high NLRX1 expression positively correlated with HIV-1 viremia in patients (107), or conversely low expression was associated to low prognosis of hepatocellular carcinoma (108) and gastric cancer (109). Similarly, expression of NLRX1 was reduced in aneurysm-induced brain injury (27) and in chronic obstructive pulmonary disease (COPD) (110). Interestingly, in COPD, disease prevalence does not seem to differ between men and women, however the clinical presentation is different and more severe in women (111). Whether NLRX1 contributes to sex differences observed in these pathologies remains to be determined. Taken together, NLRX1 represents a promising therapeutic target as a regulator of inflammation as already shown in several mice models (112–114). However, only the research approaches that consider both sexes will provide a complete understanding of the regulation of inflammation and metabolism and provide new insights for sex-specific drug development.

## Data Availability Statement

The data presented in this study are deposited in NCBI’s Gene Expression Omnibus (GEO) repository and are accessible through GEO Series accession numbers GSE201120 and GSE201066.

## Ethics Statement

The animal study was reviewed and approved by Swiss Federal Veterinary Office (SFVO).

## Author Contributions

TS and NF designed the study. TS performed experiments, analyzed the data, and wrote the first draft of the manuscript. SC, CD, FP, NI, and FT performed experiments. AB, IX, NI, FT, and CG analyzed data. IL, LF, AB, IX, SC, and NF interpreted and discussed the data. NF reviewed and edited the manuscript. All authors contributed to the article and approved the submitted version.

## Funding

This work was funded by the grants from the Swiss National fund for research to NF (Grant No. 310030_173180, and IZRJZ3_164176/1) and by Fondation Pierre Mercier pour la science.

## Conflict of Interest

The authors declare that the research was conducted in the absence of any commercial or financial relationships that could be construed as a potential conflict of interest.

## Publisher’s Note

All claims expressed in this article are solely those of the authors and do not necessarily represent those of their affiliated organizations, or those of the publisher, the editors and the reviewers. Any product that may be evaluated in this article, or claim that may be made by its manufacturer, is not guaranteed or endorsed by the publisher.
