# Supplementary material for: Sex-Biased Control of Inflammation and Metabolism by a Mitochondrial Nod-Like Receptor
Source: Front Immunol. 2022 May 16;13:882867. doi: 10.3389/fimmu.2022.882867 (PMC9150262; doi:10.3389/fimmu.2022.882867)
Supplement: Supplementary file 1 [file DataSheet_1.pdf]

Supplementary Material

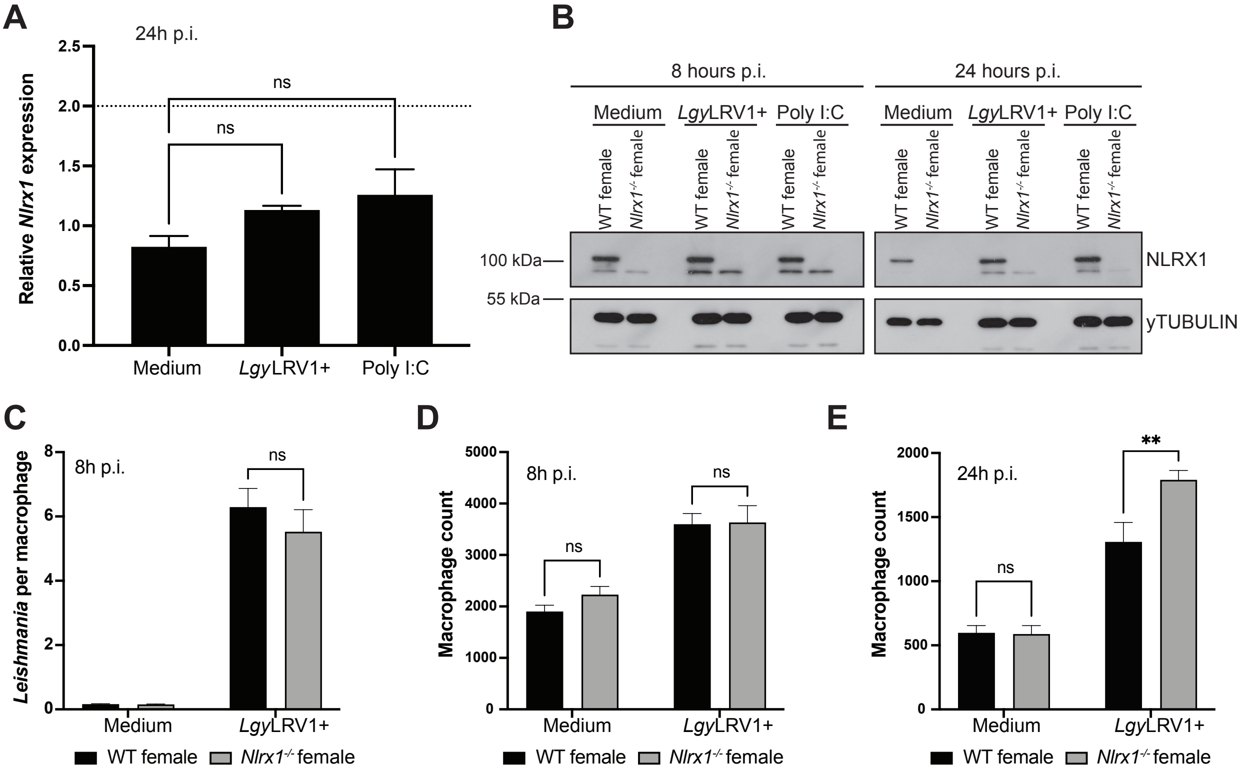

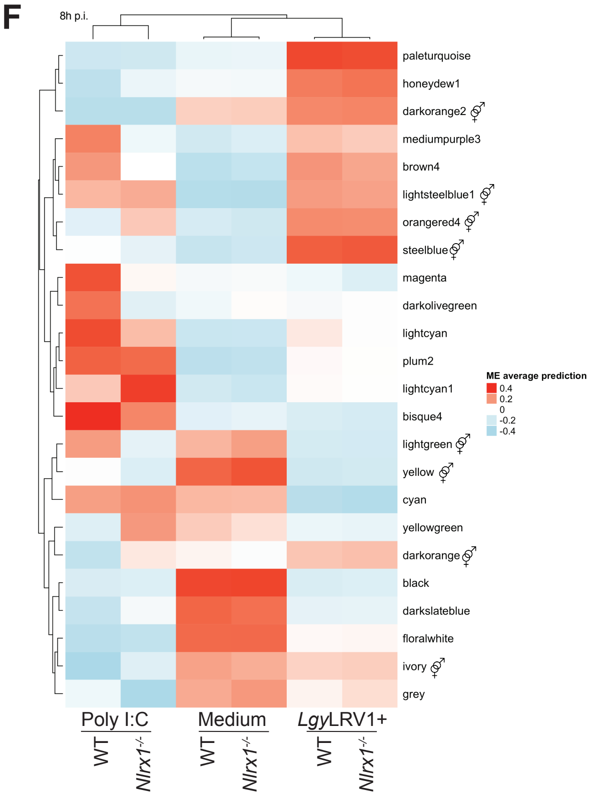

G

Timepoint	Inflammation and Infectivity	Mitochondria and Metabolism	Sex hormone signalling
**8h**	20/23 (87%)	23/23 (100%)	8/23 (34.8%)
**24h**	18/22 (81.8%)	21/22 (95.5%)	10/22 (45.5%)
Keywords	NF-kappaB<br>JAK-STAT<br>Stat<br>p38MAPK<br>JUN<br>JNK<br>interleukin<br>chemokine<br>interferon<br>phagocytosis<br>exocytosis<br>endocytosis<br>angiogenesis<br>chemotaxis<br>endosome<br>autophagosome<br>oxidative stress<br>hypoxia<br>pathogen recognition<br>toll-like<br>antigen<br>complement<br>apoptotic signalling<br>viral<br>lipopolysaccharide<br>leukocyte	mitochondria<br>cristae<br>mitophagy<br>autophagy<br>fission<br>glycolytic<br>oxidative phosphorylation<br>tricarboxylic acid cycle<br>respiration<br>ATP<br>cAMP<br>TOR<br>biosynthetic process<br>metabolic process<br>catabolic process<br>homeostasis<br>calcium<br>reactive oxygen species<br>cytochrome c<br>lipid	hormone<br>endocrine<br>steroid hormone receptor<br>estrogen<br>androgen<br>X chromosome

Supplementary Figure S1. NLRX1 modulated inflammation, infection, and metabolism in female BMDMs. BMDMs from WT and *Nlrx1*$^{-/-}$ female mice were infected with *Lgy*LRV1+ parasites or stimulated with poly I:C. 2 µg/ml). **(A)** *Nlrx1* mRNA levels were quantified by qRT-PCR at 24 hours p.i. (n=3 independent experiments). **(B)** After 8 and 24 hours, proteins were extracted blotted for NLRX1 and γ-TUBULIN. Representative western blot of 3 independent experiments is shown. **(C)** Intracellular parasite load (8 hours p.i.) and **(D-E)** macrophage count (8 and 24 hours p.i.) were quantified (n=2 independent experiments). Transcriptomics analysis of WT and *Nlrx1*$^{-/-}$ female BMDMs (n=3 mice per group) infected with *Lgy*LRV1+ parasites or stimulated with poly I:C (2 µg/ml) for 8 and 24 hours. **(F)** The heatmap represents the global weighted correlation network analysis (wgcna) at 8 hours pi. and module names are represented by a color. **(G)** A gene ontology (GO) enrichment analysis for each module was performed to identify the biological processes associated to each module and terms were grouped into 3 categories. ⚥ represents modules enriched in GO terms associated to sex hormone signaling. Graphs are presented as mean +- SEM and significance was tested by two-way ANOVA with multiple comparisons (B-D). ns = non-significant, **$p \leq 0.01$.

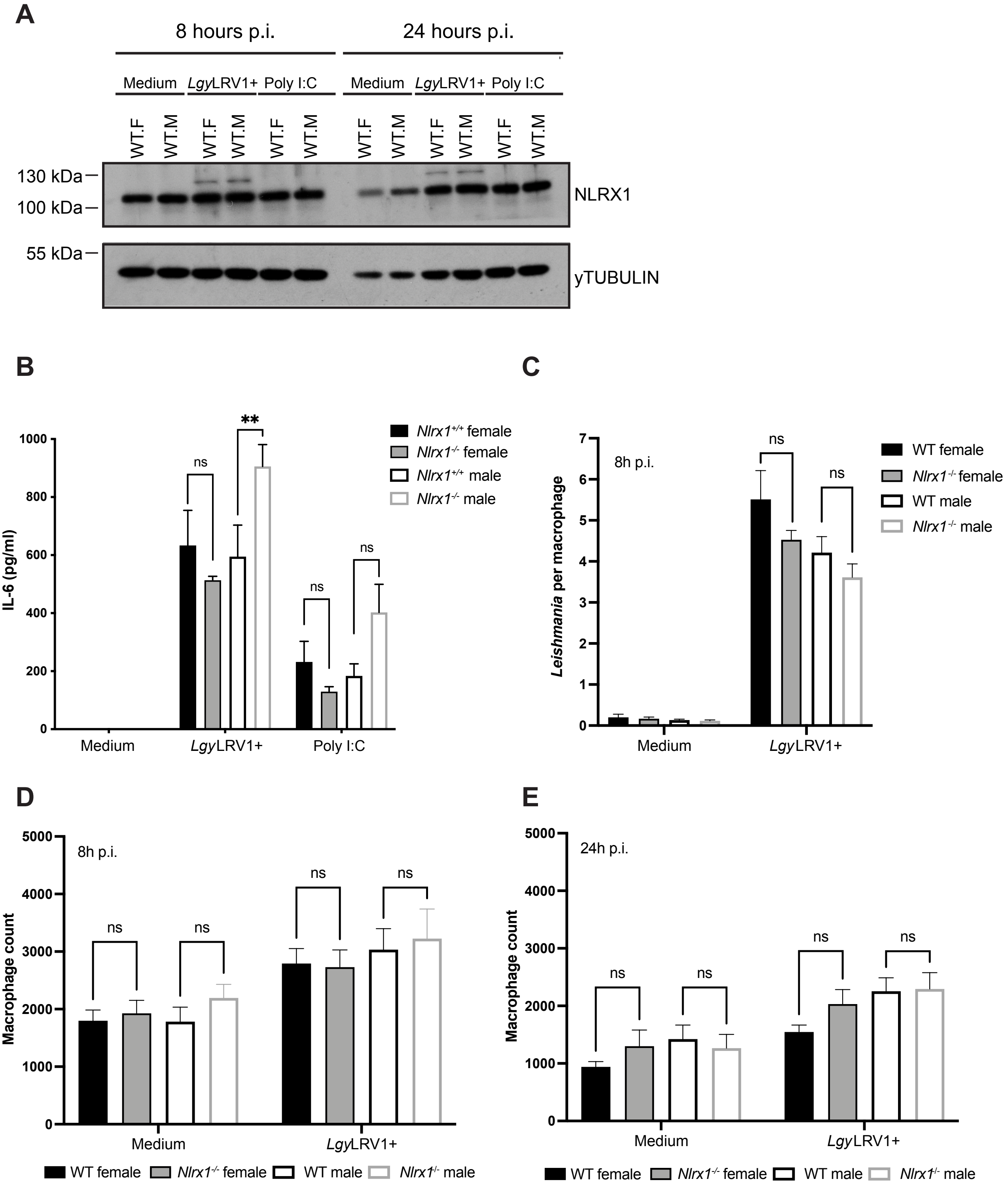

Supplementary Figure S2. Inflammation and infection in female and male macrophages. BMDMs from WT and *Nlrx1-/-* female mice were isolated and infected with *Lgy*LRV1+ parasites

or stimulated with the TLR3 agonist poly I:C (2 μg/ml). **(A)** After 8 and 24 hours, proteins were extracted from cell lysates and blotted for NLRX1 and γ-TUBULIN. WT.F, WT female. WT.M, WT male. Representative western blot of three independent experiments is shown. **(B)** As a control, after 24 hours, IL-6 was measured in in cell-free supernatants by ELISA in *Lgy*LRV1+ infected or poly I:C stimulated BMDMs isolated from littermate controls (representative experiment shown, n=3). At 8 and 24 hours p.i., BMDMs were fixed with 4% PFA and stained with DAPI and phalloidin. Cells were visualized with a high content microscope (40x) and **(C)** intracellular parasite load (8 hours p.i.) and **(D-E)** macrophage count (8 and 24 hours p.i.) were quantified using a MetaXpress software (n=3 independent experiments). Graphs are presented as mean +- SEM and significance was tested by two-way ANOVA with multiple comparisons (B-D). ns = non-significant.

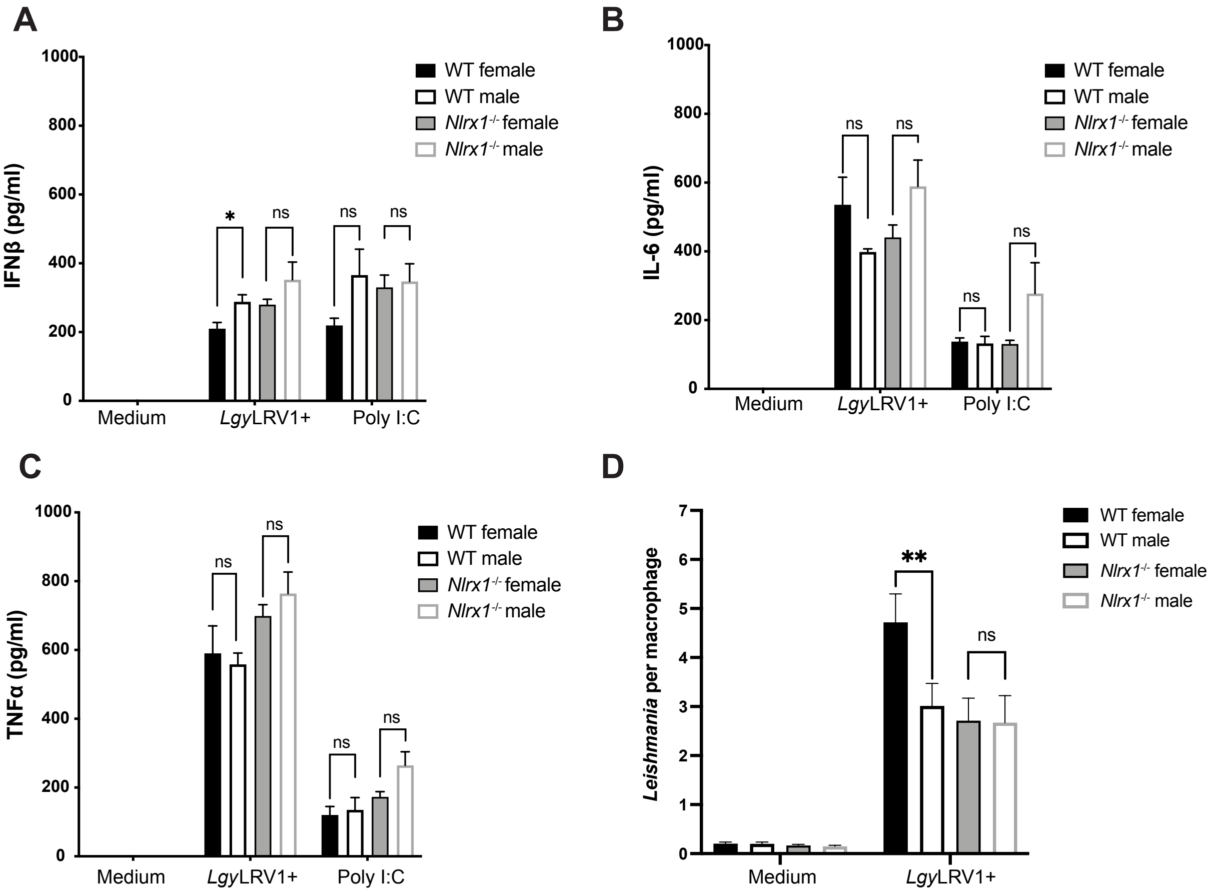

Supplementary Figure S3. Statistical analysis of inflammation and infection profiles by sex. To complement Figure 4, the statistical analysis for figures 4C-F was also performed by sex. Graphs are presented as mean +- SEM and significance was tested by two-way ANOVA with multiple comparisons (A-F). ns = non-significant, $*p \leq 0.05$, $**p \leq 0.01$.

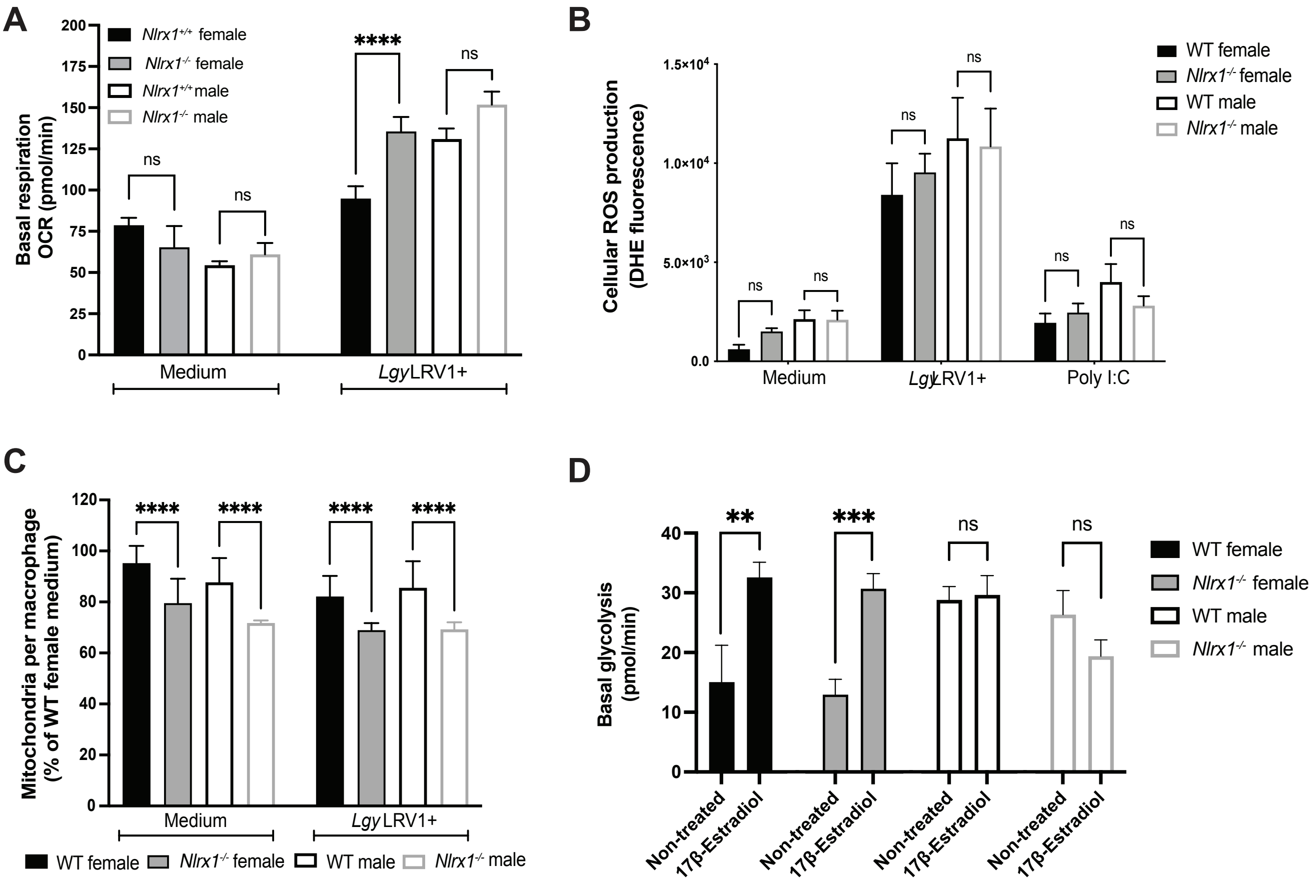
A
Nlrx1+/+ female
Nlrx1-/- female
Nlrx1+/+ male
Nlrx1-/- male
Basal respiration OCR (pmol/min)
200
175
150
125
100
75
50
25
0
ns
ns
****
ns
Medium
Lgy LRV1+
B
WT female
Nlrx1-/- female
WT male
Nlrx1-/- male
Cellular ROS production (DHE fluorescence)
1.5×10⁴
1.0×10⁴
5.0×10³
0.0
ns
ns
ns
ns
ns
ns
Medium
Lgy LRV1+
Poly I:C
C
Mitochondria per macrophage (% of WT female medium)
120
100
80
60
40
20
0
****
****
****
****
Medium
Lgy LRV1+
WT female
Nlrx1-/- female
WT male
Nlrx1-/- male
D
Basal glycolysis (pmol/min)
40
30
20
10
0
**
***
ns
ns
WT female
Nlrx1-/- female
WT male
Nlrx1-/- male
Non-treated
17β-Estradiol
Non-treated
17β-Estradiol
Non-treated
17β-Estradiol
Non-treated
17β-Estradiol

Supplementary Figure S4. Genotype- or sex-dependent effects on ROS, mitochondria, and metabolism. (A) As a control, after 8 hours, basal mitochondrial respiration rate was assessed in BMDMs isolated from littermates (representative experiment shown, n=3). **(B)** Cellular ROS (ROS) production was quantified in female and male BMDMs infected with *Lgy*LRV1+ parasites or treated with poly I:C (2 µg/ml) for 8 hours. Cells were stained with DHE (5 µM) for 20 min at 37°C. Fluorescence was measured using a Spectramax i3 plate reader and adjusted to protein concentration per well. (n=3 independent experiments). **(C)** Number of mitochondria per female and male BMDMs infected with *Lgy*LRV1+ parasites for 8 hours were counted in images acquired by transmission electron microscopy (n=2 independent experiments, total of minimum 60 cells were analyzed per group). **(D)** BMDMs from female and male WT and $Nlrx1^{-/-}$ mice were pre-treated with 17β-estradiol (200 pg/ml) for 2 hours and estradiol was kept in the assay medium for the duration of the assay. Basal glycolytic rate was assessed by Seahorse XFe96 analyzer and adjusted to protein concentration per well (n=4 independent experiments). Graphs are presented as mean +- SEM and significance was assessed by two-way ANOVA with multiple comparisons (A) or unpaired, parametric t-test (B-C). ns: non-significant, $**p \leq 0.01$, $***p \leq 0.001$, $****p \leq 0.0001$.

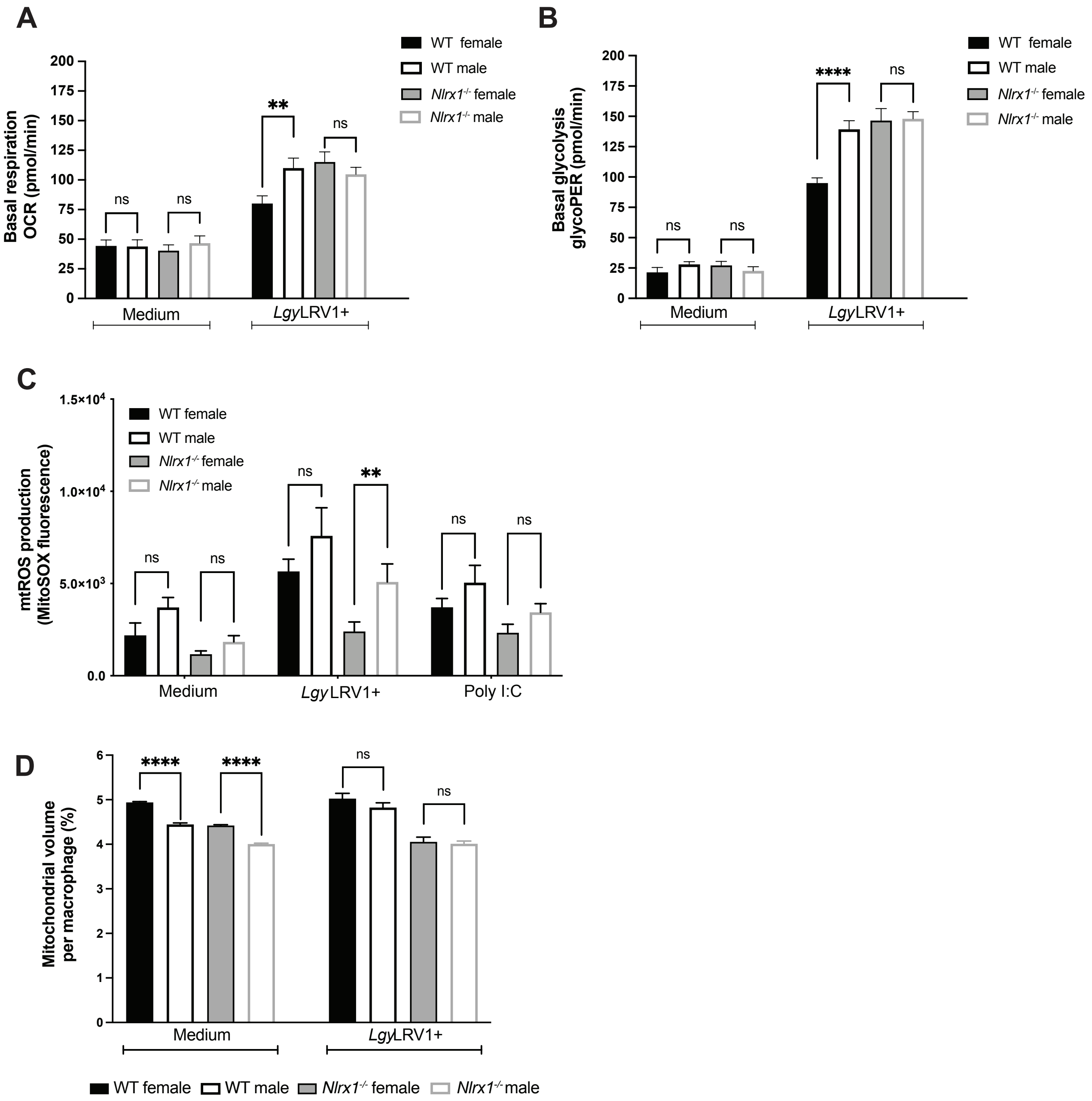

Supplementary Figure S5. Statistical analysis of metabolic profiles by sex. To complement Figure 5, the statistical analysis for figures 5A-C and figure 5E was also performed by sex. Graphs are presented as mean +- SEM and significance was assessed by two-way ANOVA with multiple comparisons (A-C) or unpaired, parametric t-test (D). ns = non-significant, $^{**}p \leq 0.01$, $^{****}p \leq 0.0001$.

A

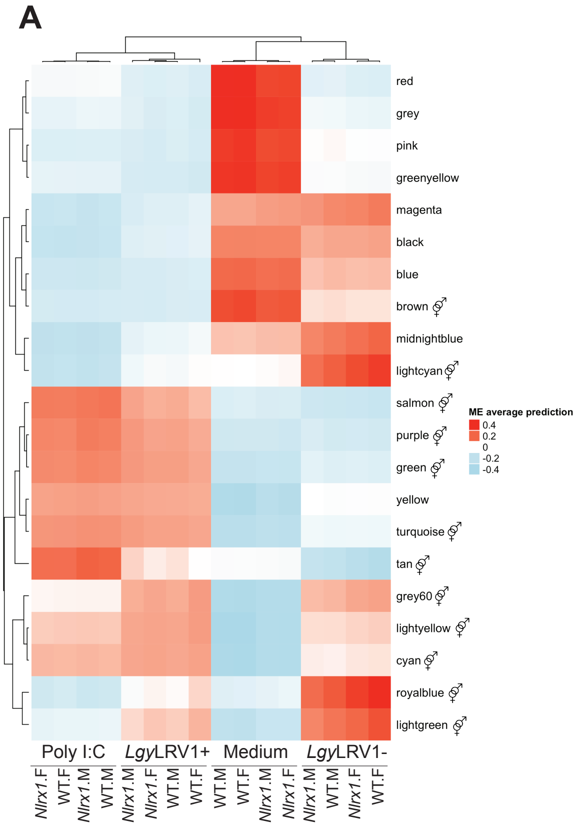

B

Timepoint	Inflammation and Infectivity	Mitochondria and Metabolism	Sex hormone signalling
8h	20/20 (100%)	19/20 (95%)	12/20 (60%)
24h	6/6 (100%)	5/6 (83.3%)	5/6 (83.3%)
Keywords	immunity<br>immune response<br>NF-kappaB<br>JAK-STAT<br>p38MAPK<br>JNK<br>interleukin<br>chemokine<br>interferon<br>phagocytosis<br>exocytosis<br>endocytosis<br>angiogenesis<br>chemotaxis<br>xenophagy<br>endosome<br>autophagosome<br>oxidative stress<br>hypoxia<br>toll-like<br>antigen<br>complement<br>apoptotic signalling<br>viral<br>lipopolysaccharide<br>leukocyte	mitochondria<br>fission<br>mitophagy<br>autophagy<br>glycolytic<br>oxidative phosphorylation<br>tricarboxylic acid cycle<br>ATP<br>biosynthetic process<br>metabolic process<br>catabolic process<br>homeostasis<br>calcium<br>reactive oxygen species<br>lipid	hormone<br>steroid hormone receptor<br>estradiol<br>estrogen<br>androgen<br>testosterone<br>endocrine

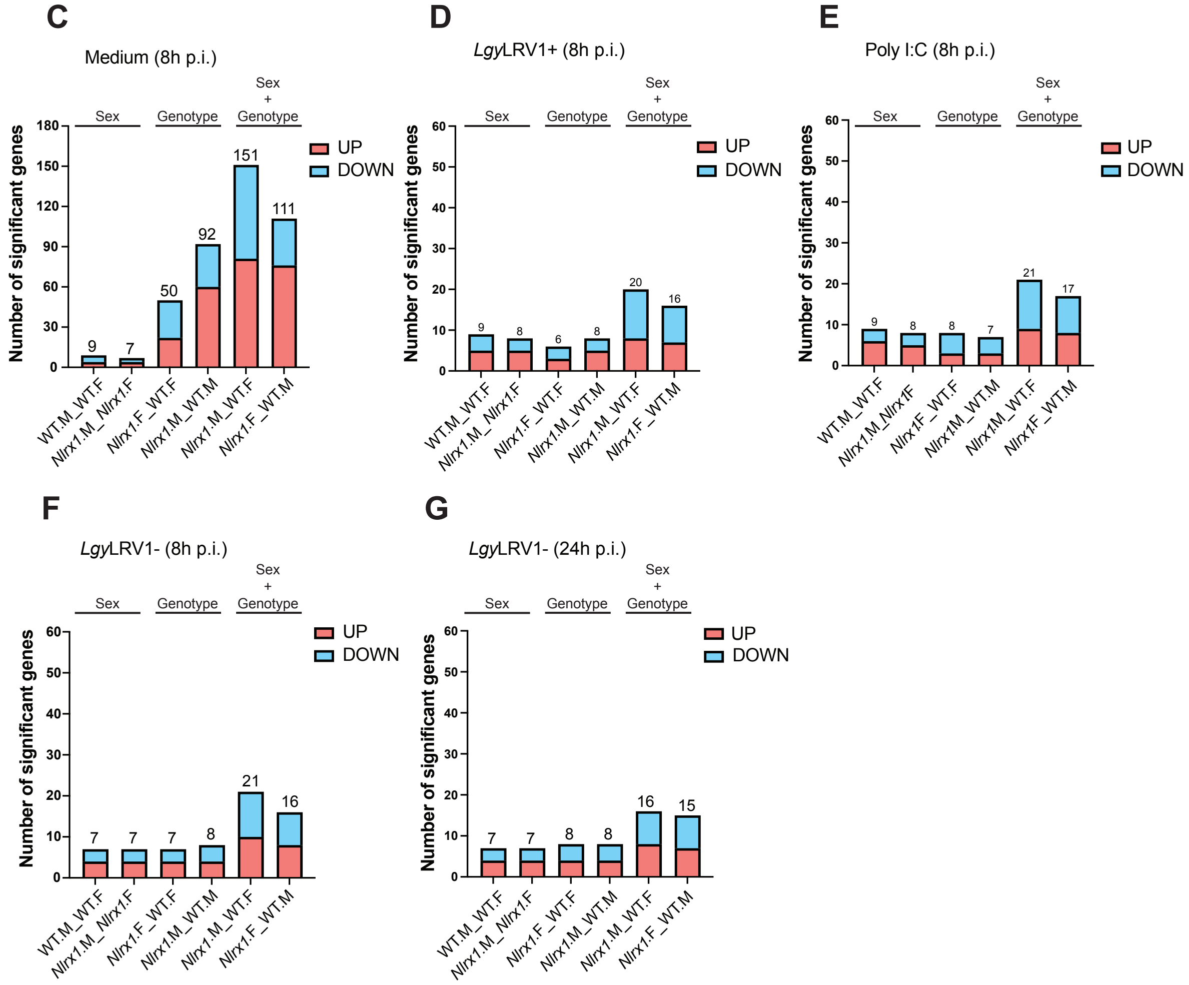

Supplementary Figure S6. Transcriptomics analysis of female and male BMDMs. Transcriptomics analysis of both female and male WT and *Nlrx1*$^{-/-}$ BMDMs (n=3 mice per group) infected with *Lgy*LRV1+ parasites or stimulated with poly I:C (2 µg/ml) for 8 hours. **(A)** The heatmap represents the global weighted correlation network analysis (wgcna) of 8 hours pi. and module names are represented by a color. A gene ontology (GO) enrichment analysis for each module was performed to identify the biological processes associated to each module and **(B)** terms were grouped into 3 categories. ⚥ represents modules enriches in GO terms associated to sex hormone signaling. At 8 hours, the number of differentially expressed genes between groups in **(C)** non-infected, **(D)** *Lgy*LRV1+ infected and **(E)** poly I:C treated conditions, and **(F-G)** at 8 and 24 hours, in LgyLRV1- infected conditions the number of differentially expressed genes between groups is plotted. On each barplot the number represents the total number of differentially upregulated (in red) and downregulated (in blue) genes. Pairwise comparisons are done by "sex" (same genotype, different sex, "WT.F._WT.M" and "*Nlrx1*.F_*Nlrx1.M"),* by "genotype" (same sex, different genotype ("*Nlrx1*.F_WT.F" and "*Nlrx1*.M_WT.M"), or by combining both "sex and genotype" ("*Nlrx1*.M_WT.F" and "*Nlrx1*.F_WT.M"). See **Supplementary tables 12-16** for the list of significantly differentially expressed genes.
